# Recent Advances of Graphene Quantum Dots in Chemiresistive Gas Sensors

**DOI:** 10.3390/nano13212880

**Published:** 2023-10-30

**Authors:** Xiaofeng Zhu, Yongzhen Li, Pei Cao, Peng Li, Xinzhu Xing, Yue Yu, Ruihua Guo, Hui Yang

**Affiliations:** 1 School of Materials Science and Engineering, Zhejiang University, Hangzhou 310027, China; zhuxiaofeng@bjast.ac.cn; 2 Institute for Smart Ageing, Beijing Academy of Science and Technology, Beijing 100089, China; liyongzhen@bjast.ac.cn (Y.L.); caopei2022@163.com (P.C.); lipeng2023@bjast.ac.cn (P.L.); xingxinzhu@bjast.ac.cn (X.X.); yuyue@bjast.ac.cn (Y.Y.)

**Keywords:** graphene quantum dots (GQDs), nanocomposites, chemiresistive, gas sensor

## Abstract

Graphene quantum dots (GQDs), as 0D graphene nanomaterials, have aroused increasing interest in chemiresistive gas sensors owing to their remarkable physicochemical properties and tunable electronic structures. Research on GQDs has been booming over the past decades, and a number of excellent review articles have been provided on various other sensing principles of GQDs, such as fluorescence-based ion-sensing, bio-sensing, bio-imaging, and electrochemical, photoelectrochemical, and electrochemiluminescence sensing, and therapeutic, energy and catalysis applications. However, so far, there is no single review article on the application of GQDs in the field of chemiresistive gas sensing. This is our primary inspiration for writing this review, with a focus on the chemiresistive gas sensors reported using GQD-based composites. In this review, the various synthesized strategies of GQDs and its composites, gas sensing enhancement mechanisms, and the resulting sensing characteristics are presented. Finally, the current challenges and future prospects of GQDs in the abovementioned application filed have been discussed for the more rational design of advanced GQDs-based gas-sensing materials and innovative gas sensors with novel functionalities.

## 1. Introduction

Currently, with the vigorous development of the Internet of Things (IoTs), gas sensing technology has received a lot of attention in various fields [1,2,3], including environment assessment, medical diagnostics, industrial safety, automobile exhaust detection, and explosive gas monitoring, which is vital for intelligent life in the future [4,5]. According to the characteristics of an ideal gas sensor [6,7], different types of gas sensors with various operating principles have been well designed and investigated, e.g., electrochemical [8,9,10], optical [11,12], chemiresistive [13,14,15,16,17,18], and field-effect transistor-type (FET-type) [19,20] gas sensors. Among them, the chemiresistive gas sensors have attracted great interest because of their mature fabrication methods, simple sensing structures, small amounts of active materials, widely used sensitive materials, wide detecting range of gases, compatibility with modern electronic devices, and low cost [21,22,23]. The changes in surface resistance of the sensing layer during the adsorption and desorption of the target analyte form the foundation for the receptor and sensing functions of chemiresistive gas sensors [24,25]. The sensitive materials and the structures of sensing layers possessing remarkable properties are the core components of gas sensors [1].

The sensing materials utilized in the fabrication of chemiresistive gas sensors mainly consist of semiconducting metal oxide nanostructures (SMONs) [16,18,26,27,28], conducting polymers [29], two-dimensional (2D) materials [30,31], carbon-based nanomaterials [32,33,34,35,36], and transition metal carbides and nitrides (MXenes) [37,38,39]. Among these, SMONs have been widely studied and used as gas sensors in multidisciplinary applications because of their excellent physical and chemical properties [16,18,27], but it is still a huge challenge to break the performance bottlenecks including low working temperature, trace gas detection, and excellent selectivity in the practical applications [40,41]. Thus, enormous efforts have been made to improve the sensing capability of SMONs sensors by doping [42,43,44], incorporation of heterostructures [15,45,46,47,48], novel-morphology nanostructure design [26,49,50], and functionalization modification [51,52,53,54]. Conducting polymers and their derivatives suffer from low thermal stability and low sensitivity, leading to making the polymer hybrids with metal oxides [55,56].

Carbon-based nanomaterials have been at the forefront of materials science for the past few decades, which as sensitizers can effectively improve the sensitivity of gas sensors or reduce their operating temperature [57,58,59,60]. Recently, graphene and graphene oxide (GO), due to their large specific surface area, tunable crystal defect density, high thermal and electrical conductivity, carrier mobility, and mechanical strength, have been recognized as promising candidates for gas sensing materials [61,62,63]. Reduced graphene oxide (rGO), shows more dangling bonds and structural defects than pure GO, providing favorable conditions for gas adsorption [64,65]. However, sensors based on pure rGO typically have low gas sensitivity to analytes at room temperature [66]. In addition, owing to few defects or functional groups on the original graphene surface or edges, it cannot provide adsorption sites for gas molecules [67,68] and the huge surface of graphene can prevent target gas molecules from reaching to sensing materials [69]. Therefore, low sensitivity, long response/recovery time, poor selectivity, dispersion, and easy aggregation of graphene/GO/rGO based materials limit their further development in gas sensing applications [70].

Graphene quantum dots (GQDs), as a 0D nanomaterial, have distinct graphene lattices, characterized by atomic thin graphite planes (usually 1 or 2 layers, with a thickness < 2 nm), and lateral dimensions typically < 10 nm [71,72]. Unlike 2D graphene, GQDs combine the properties of the graphene and the advantages of QDs, which are related to strong quantum confinement, edge effects, and adjustable electronic band structures, leading to novel and extraordinary applications in the fields of sensors, solar cells, photovoltaic devices, batteries, bio-imaging, drug delivery, etc. [73,74,75,76,77,78,79,80]. GQDs with defective structures, excellent electronic mobility, minute size, high specific surface area, and abundant functional groups can have effective adsorption sites and provide a better driving force for gas diffusion, and simultaneously be further functionalized with various inorganic substances and easily be introduced into other functional materials to form high-quality nanostructured materials, which can remarkably enhance the gas-sensitivity towards the analytes [72,81,82,83,84,85,86]. Moreover, the semiconducting properties (n- or p-type) of GQDs can be adjusted by doping with heteroatoms [87,88,89] and the electronic structure of GQDs can be tuned over by changing their size over a wide energy range, which is essential to achieve selectivity in a variety of target applications. As we all know, the sensing mechanism of sensitive materials is based on the reaction between oxygen adsorbed on the surface of the material and the detected gas molecules [84]. The state and quality of oxygen on the surface of materials largely depend on the specific surface area, microstructure, and particle size of the sensing material [84]. The numerous carboxylic groups (carbonyl and hydroxyl groups) can produce oxygen-rich content in the lattices of GQDs, leading to a high sensor response.

There have been many excellent reviews on GQDs in fluorescence-based ion sensing, biosensing, bioimaging, and electrochemistry, photo-electrochemistry and electrochemiluminescence sensing, as well as therapeutic, energy, and catalysis applications [59,72,90,91,92,93,94,95,96], but there is still no single review article on the application of GQDs in the field of chemiresistive gas sensing. This is our primary inspiration for writing this review, with a focus on chemiresistive gas sensors reported using GQDs and its composites. So far, metal oxide/GQDs composites, metal-metal oxide/GQDs composites, ternary metal oxide/GQDs composites, bimetallic oxide/GQDs composites, metal phthalocyanine (MPc)/GQDs, polymer/GQDs, metal oxide/polymer/GQDs composites, metal sulfide/GQDs composites, and GQDs/SiNW have been reported to be used for sensing various gases, such as formaldehyde (HCHO), acetone, ammonia (NH_3_), hydrogen sulfide (H_2_S), isopropanol, methanol, trimethylamine, acetic acid gas, benzene, dimethyl methylphosphonate (DMMP) gas, nitrogen dioxide (NO_2_), and nitric oxide (NO). However, the number of chemiresistive gas sensors reported using GQDs and their composites is still limited. In this paper, the recent advances in GQDs-based chemiresistive gas sensors have been comprehensively summarized from the aspects of the synthesized strategies of GQDs and their composites, gas sensing enhancement mechanisms, and the resulting sensing characteristics. Finally, a summary and future perspectives are proposed on the rational design of advanced gas sensing materials based on GQDs and innovative gas sensors with novel functionalities.

## 2. Synthesis of GQDs and Their Composites

Material synthesis is the main and critical method before materials are used in specific applications. The effective synthetic strategies of GQDs and their composites, giving them specific properties, shape, size, surface characteristic, and inner structure, opens an efficient pathway to develop a high-performance gas sensor. The properties and distribution of GQDs and their composites have been shown to vary depending on their synthesis methods, reaction conditions, as well as the precursor materials used [97]. In this section, a brief outlook on the synthesis process of GQDs and their composites reported for chemiresistive gas-sensing applications is provided and presented in Table 1 and Table 2.

### 2.1. Synthesis of GQDs

**Figure 1:**
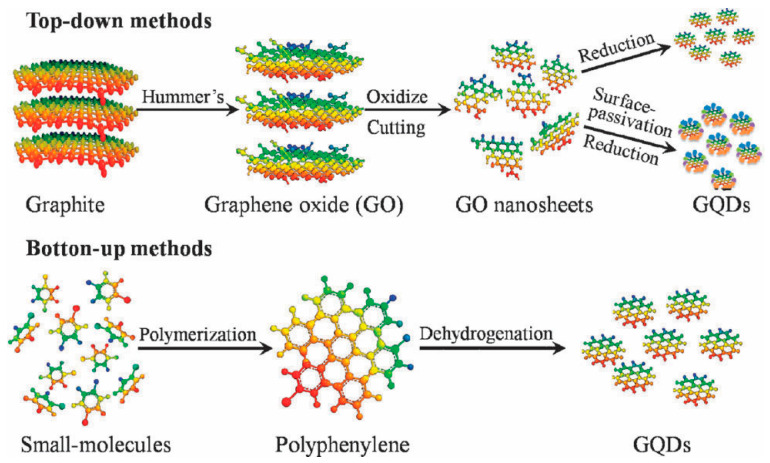
Schematic diagram of top-down and bottom-up GQDs synthesis methods (Reproduced with permission from [79]. Copyright 2012, Royal Society of Chemistry).

In general, GQDs could be synthesized through top-down and bottom-up approaches (Figure 1) [59,72,90,91,92,93,94,95,124]. In the top-down approaches, the cheap, readily available bulk graphitized carbon materials, such as graphite, graphene, GO, rGO, CNTs, and carbon fibers, were cut down into small pieces of graphene sheet by a series of chemical or physical treatments, which were carried out under harsh conditions involving concentrated acids, strong oxidizing agents, and high temperatures. Until now, chemical oxidation, hydrothermal/solvothermal processes, electrochemical exfoliation, microwave irradiation, laser ablation, and chemical exfoliation had been used to synthesize GQDs in top-down approaches. Such approaches generally afforded GQDs possessing oxygen functional groups on the surface, thus facilitating their excellent solubility and subsequent functionalization with other molecules and functional nanomaterials [91]. However, due to the randomness of the cutting sites, such methods lacked precise control of particle size distribution and morphology of GQDs [94,125]. In bottom-up approaches, GQDs were synthesized by chemical assembling of small aromatic precursors or other carbon-containing moieties under pyrolysis/hydrothermal/solvothermal conditions, carbonization of organic molecules or step-by-step organic synthesis. Comparatively, bottom-up synthesis provided excellent control over the size, morphology, shape, surface state, and properties of GQDs. However, strict reaction conditions and the correct selection of organic precursors were demanded for these methods, which could greatly influence the GQDs’ final gas sensing properties [96,126]. Elaboration is provided in the following subsections on the details of synthesis of GQDs reported for the chemiresistive gas sensors.

#### 2.1.1. Top-Down Approaches

**Figure 2:**
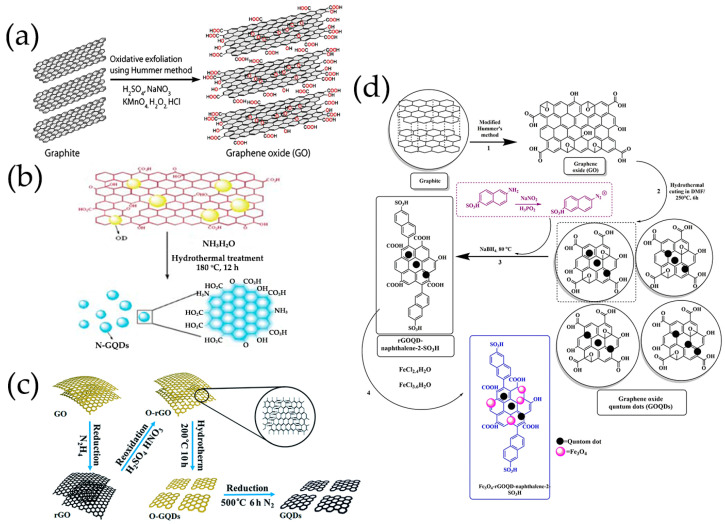
(**a**) Schematic representations of the modified Hummers method; (**b**) Schematic illustration of the preparation of N-GQDs through GO hydrothermal treatment in the presence of ammonia; (**c**) Schematic diagram for the preparation process of GQDs; (**d**) Synthesis of functionalized rGOQDs composite. Reproduced with permission from [58,104,127,128]. Copyright 2015, American Chemical Society; copyright 2013, 2021, Royal Society of Chemistry; copyright 2021, Springer.

The first step in many methods was to convert graphite-based precursors to graphite oxide or graphene oxide, in which a modified Hummers method was most commonly used [58,70,98,99,100,101,105,125] (Figure 2a,d). This method used a mix of H_2_SO_4_, NaNO_3_, and KMnO_4_ or similar reagents [125]. Then, reductive agents (e.g., hydrazine hydrate [98,102], ammonia [70,99,100], and DMF [103,104]) cleaved GO into GQDs by breaking the C-O bond in the epoxy groups under hydrothermal/solvothermal conditions (Figure 2b–d). Reduction cracking introduced nitrogen-containing chemical groups and reduced oxygen-containing groups [72]. Jiang et al. [58,101] made GQDs from GO involving several steps, namely, reduction of GO with hydrazine to rGO, reoxidation of rGO in H_2_SO_4_ and HNO_3_ to O-rGO, hydrothermal cutting of O-rGO to O-GQDs, and then reduction of O-GQDs to afford the GQDs (Figure 2c). In hydrothermal/solvothermal methods, the principle was to break the bonds between GO under high pressure and high temperature to generate GQDs [90].

The electrochemical exfoliation of rGO film [105] and graphite rods [106] could produce single layer uniform GQDs with at high yield, which did not require the use of strong acids or high-temperature and high-pressure conditions. It has the advantages of low-cost, reproducibility, environmental friendliness, and simple operating principles. Li et al. [105] reported a three-step method for converting graphite powder into GQDs, with a free-stranding rGO film as the working electrode and PBS as the electrolyte. Wongrat et al. [106] synthesized GQDs utilizing graphite rods as electrodes in citric acid and KCl electrolyte.

Liquid-phase exfoliation was a unique ultrasonic assisted process. During this process, numerous bubbles generated in the carbon-based precursors generated mechanical forces that destroyed internal carbon bonds, resulting in the formation of GQDs [90]. Chen et al. [68] and Liu et al. [107] reported, respectively, the synthesis of GQDs via an ultrasonic-assisted exfoliation of MWCNTs and graphite powder in H_2_SO_4_ and HNO_3_. The pH value of GQDs obtained from MWCNTs could be further adjusted to obtain acidic and neutral NH_3_ gas sensors. Using graphite powder [108] and degreasing cotton [109] as precursors, Zhang et al. synthesized element-doped GQDs. Boron (B) and oxygen functional groups were incorporated into the layered graphite powder by boric acid treatment, and then treated under ultrasonication to get B-GQDs [108]. Carbon fibers (CF) were fully carbonized from degreasing cotton at 1000 °C, and then treated with boric acid, HNO_3_, and HCl under ultrasonication to get B, N, and Cl-doped GQDs, respectively [109]. Zhang et al. [109] also reported the physical grinding synthesis of S-GQDs using graphite and sulfur as the precursors. The precursors were placed in an agate jar containing agate balls and fixed in a planetary ball mill.

#### 2.1.2. Bottom-Up Approaches

**Figure 3:**
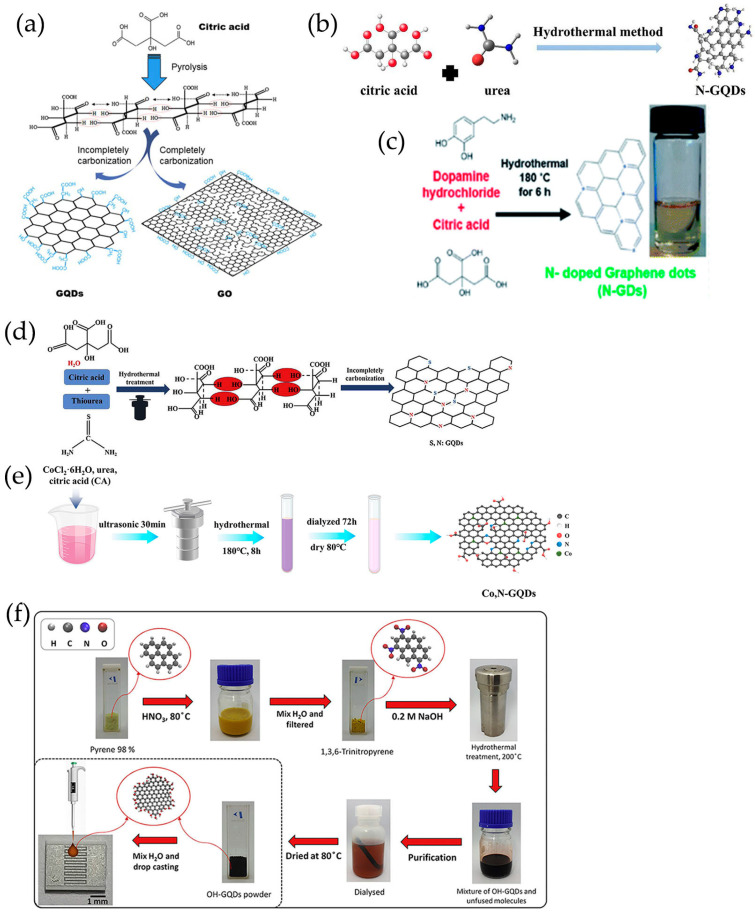
(**a**) Synthesis process of GQDs and GO; (**b**) Synthesis process of N-GQDs; (**c**) Schematic of the synthesis of N-GQDs; (**d**) The preparation process of S, N-GQDs; (**e**) Schematic illustration of the synthesis of Co, N-GQDs; (**f**) Schematic diagram of OH-GQDs synthesis and the fabrication process of gas sensors based on OH-GQDs (**bottom left**). Reproduced with permission from [114,118,120,121,129]. Copyright 2012, 2021, Elsevier; copyright 2020, Royal Society of Chemistry; copyright 2019, 2020, Elsevier.

The most commonly used bottom-up methods for synthesizing GQDs as chemiresistive gas-sensing platforms were pyrolysis and hydrothermal synthesis, in which citric acid (CA) had been widely used as the carbon source by various research groups (Table 1). Dong et al. [129] developed a simple synthesis method to synthesize GQDs by direct pyrolysis of CA using a heating mantle device (Figure 3a). Formation of intermolecular hydrogen bonds and subsequent dehydration reactions generated the sp^2^ carbon networks [72,129]. Note that, when the reaction time was extended, GO was generated instead of GQDs [72,129] (Figure 3a). Then, the resultant GQDs formed composites with α-Fe_2_O_3_ [84] and ZnO [69] and created nanofeatures on the microchannel’s wall [86] for sensing trimethylamine [84], ethanol [69], and VOCs [86], respectively.

Precursor molecules treated by hydrothermal methods had been demonstrated to be another effective method for producing GQDs, indicating that heteroatoms were easily doped during the synthesis process. Chu et al. [110,111] synthesized GQDs through one-step hydrothermal treatment with citric acid. Using urea [112,113,114,115,116,117] and dopamine hydrochloride [118] as N source, thiourea as S and N source [67,120] respectively, N-doped GQDs (N-GQDs), and S, N co-doped GQDs (S, N-GQDs) were obtained in the presence of CA (Figure 3b–d). In our group, cobalt (Co) and N co-doped GQDs [119] had been successfully prepared by using CA, urea, and CoCl_2_·6H_2_O as precursors (Figure 3e). In addition to CA, some small polycyclic aromatic hydrocarbons (PAHs) similar to graphene could also be used as precursors for GQDs [130]. The nitrated pyrene [121] and benzopyrene [122] had been used to synthesize GQDs via hydrothermal method (Figure 3f).

### 2.2. Synthesis of GQDs-Based Composites

#### 2.2.1. Metal Oxide/GQDs Composites

**Figure 4:**
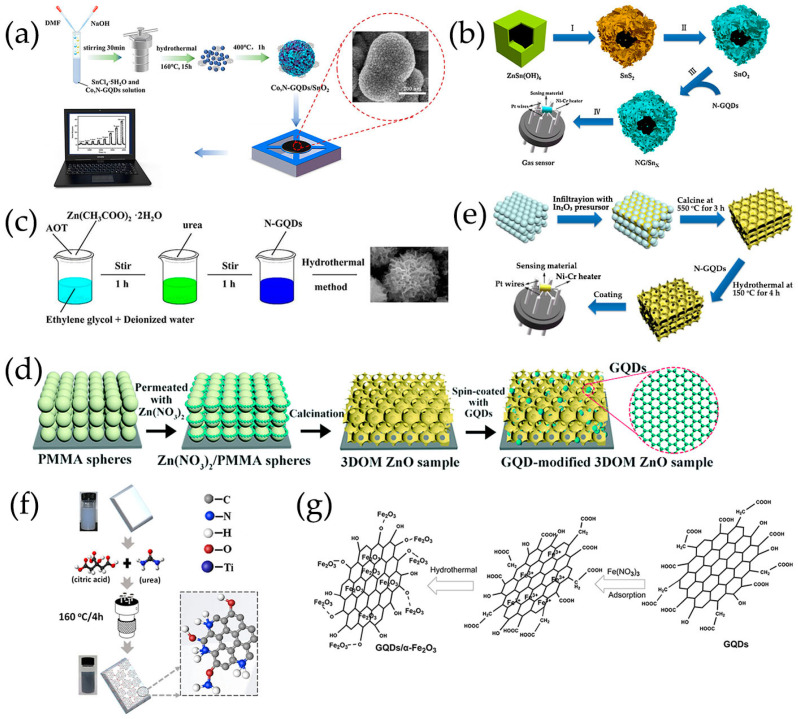
(**a**) Synthesis of Co, N-GQDs/SnO_2_ mesoporous microspheres and schematic diagram of sensor testing; (**b**) Schematic diagram of the synthesis of gas sensors based on N-GQDs/SnO_2_ hierarchical hollow cube; (**c**) Synthesis process of N-GQDs/ZnO composites; (**d**) Synthesis process of GQDs-modified 3DOM ZnO sample; (**e**) Diagrammatic illustration of N-GQDs/In_2_O_3_; (**f**) Synthesis of TiO_2_/N-GQDs; (**g**) Schematic representation of the formation process of GQDs/Fe_2_O_3_ composites. Reproduced with permission from [70,84,100,107,113,114]. Copyright 2021, Elsevier; copyright 2019, Royal Society of Chemistry; copyright 2020, American Chemical Society; copyright 2016, Elsevier.

SnO_2_/GQDs nanocomposites were synthesized by solvothermal treatment using GQDs DMF suspension, SnCl_4_·5H_2_O and urea as reactants, exhibiting good responsiveness and selectivity to acetone vapor [103]. GQDs were rich in electrons and had a large number of oxygen-containing groups such as epoxy, carbonyl, and hydroxyl on the edges of GQDs, providing a platform for electrostatic interactions between negatively charged oxide functional groups of GQDs and positively charged Sn^4+^. Sn^4+^ ions could be anchored onto GQDs through electrostatic force. In the presence of urea, SnO_2_ nanocrystals were formed from Sn^4+^ ions on the surface of GQDs during the solvothermal process [103]. In our group, Co, N-doped GQDs/SnO_2_ mesoporous microspheres were synthesized by solvothermal route, exhibiting synergistic enhancement of gas sensing characteristics for the detection of H_2_S gas (Figure 4a) [119]. Chen et al. [116,117] prepared N-GQDs/SnO_2_ with different morphology for the detection of formaldehyde, in which SnO_2_ nanosheets and 2D mesoporous ultrathin SnO_2_ were prepared via a hydrothermal route and by calcining GO and dibutyltin dilaureate at 500 °C, respectively. In order to achieve low-temperature detection of NO_2_, 0D N-GQDs/SnO_2_ heterostructures and N-GQDs modified mesoporous SnO_2_ hierarchical hollow cubes were synthesized. SnO_2_ quantum dots were synthesized using high-boiling-point organic solvents as raw materials by a simple heating method, then 0D N-GQDs/SnO_2_ heterojunctions were synthesized using a simple wet chemical method, which enabled the ultrasensitive and selective sensing of NO_2_ [118]. Lv et al. [100] successfully prepared a novel N-GQDs modified SnO_2_ hierarchical hollow cubes through a simple sulfuration-oxidation and impregnation-calcination, which was beneficial for improving the sensing performance of NO_2_ (Figure 4b).

GQDs/ZnO composites with different GQDs contents were synthesized using a hydrothermal method, which demonstrated that the content of GQDs in the composite had a major impact on the gas-sensitive response and selectivity of acetic acid gas [110] and NO_2_ (Figure 4c) [114]. GQDs were successfully modified onto ZnO surface to form heterojunctions, which showed an improved ammonia sensing at room temperature [106]. Liu et al. [107] proposed a facile self-assembled template strategy and spin-coating process to create 3D ordered macroporous (3DOM) ZnO structure functionalized with GQDs (Figure 4d), which showed excellent response to acetone and had a high potential for non-invasive real-time diagnosis of diabetes. ZnO nanorods (NR) were grown on pre-seeded glass substrates using a solvothermal method, and GQDs were dropped on the ZnO NR thin film. The incorporation of GQDs significantly enhanced the sensitivity of ZnO NR thin film to ethanol gas [69].

Lv et al. [70] (Figure 4e) prepared a series of N-GQDs/3DOM In_2_O_3_ composites exhibiting excellent NO_2_ sensing properties, in which 3DOM In_2_O_3_ was prepared using a PS template method. Composites of TiO_2_ rectangular nanoplate with exposed facets and N-GQDs were successfully synthesized by hydrothermal treatment, which showed the enhancement of NO sensing properties at room temperature under UV irradiation (Figure 4) [113]. Crystalline nanoporous GQDs/TiO_2_ thin films were constructed by hydrothermal treatment at 120 °C, and GQDs with water solubility were successfully incorporated into the thin film at the optimal density, forming a new GQDs/TiO_2_ heterojunction for detecting VOCs, especially isopropanol vapor, at room temperature [123]. Shao et al. [99] also prepared a new 3D structure gas microsensor using a facile post-synthetic hydrothermal method, in which the Au-modified N-GQDs/TiO_2_ nanoporous nanospheres were uniformly distributed over the whole surface of graphene-foam frame, showing good gas-sensing performance for ppb formaldehyde, which had certain application prospects in respiratory detection.

The GQDs/α-Fe_2_O_3_ composites prepared by one-step simple hydrothermal treatment exhibited enhanced gas sensing response and selectivity to trimethylamine (Figure 4g) [84]. The performance of a new magnetic nanocomposite of Fe_3_O_4_-rGOQD-naphthalene-2-SO_3_H sensor with different rGOQD-naphthalene-2-SO_3_H loadings was evaluated in order to detect NO_2_ gas [104]. The simple synthesis method (Figure 2d) of this newly nanocomposite sensor made it suitable for ultrasensitive detection of NO_2_ gas.

#### 2.2.2. Metal-Metal Oxide/GQDs Composites

Shao et al. [98] reported in situ preparation of GQDs/Pt-modified nanoporous SnO_2_ thin films with high crystallinity and nanostructured order on sensor devices. A precursor solution of GQDs/Pt-Sn was spin-coated onto a sensor device with an interdigital electrode to form a gas sensing film. Then, the films were exposed to a water vapor hydrothermal treatment with a relative humidity of 90% at a temperature of 120 °C for 96 h, and annealed at 300 °C for 2 h, which played a role in reversible transfer of acetone sensing behavior at room temperature.

#### 2.2.3. Ternary Metal Oxide/GQDs Composites

A similar procedure was reported by Shao et al. [102] with a slight modification. A highly sensitive and selective ordered nanoporous PtO_x_/GQDs/TiO_2_ membrane was prepared onsite using water vapor hydrothermal treatment and oxygen-plasma treatment for sensor devices at room temperature, exhibiting obvious adjustable sensing behavior for oxygen functionalized VOCs under visible light irradiation. In addition, Shao et al. [83] proposed a self-assembly strategy to create porous and hierarchical SnO_2_ quantum nanoparticle (SnO_2_QNP)/ZnO nanostructures functionalized by GQDs. Through a post-synthetic humidity treatment, SnO_2_QNP/ZnO nanosheets were directly self-assembled on digital integrated electrodes. The construction of ZnO nanosheet heterostructures loaded with GQDs and SnO_2_QNP was highly controllable and replicable, exhibiting significant high response, fast response/recovery time and excellent selectivity for H_2_S to other interfering gases.

#### 2.2.4. Bimetallic Oxide/GQDs Composites

**Figure 5:**
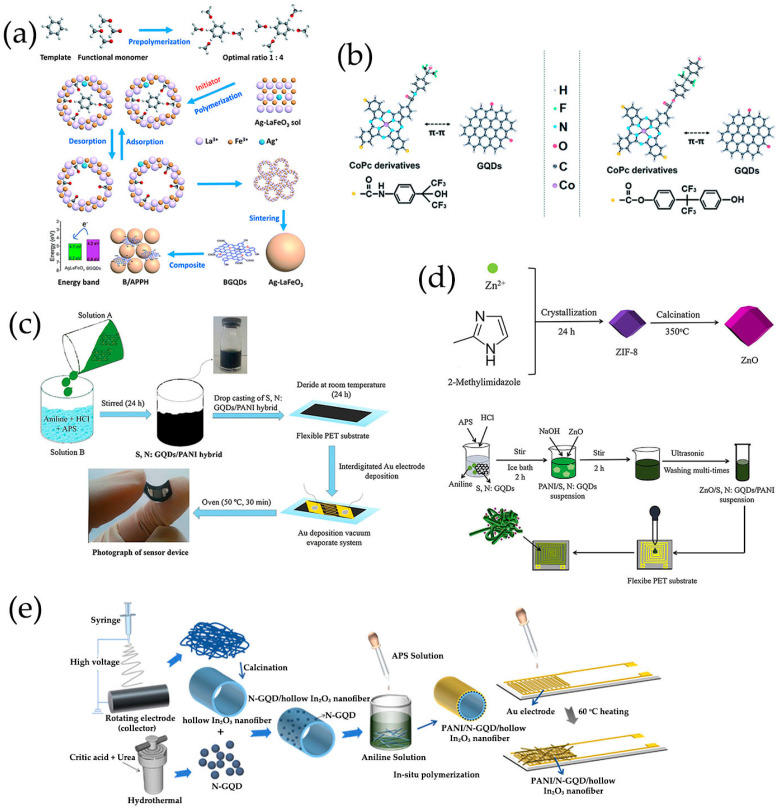
(**a**) Synthesis process of B-GQDs/Ag-LaFeO_3_; (**b**) Preparation of CoPc-HFIP/GQDs or CoPc-6FBPA/GQDs; (**c**) Schematic diagram of S, N-GQDs/PANI gas sensor fabrication process; (**d**) Preparation process of ZnO and drop-casting manufacturing of sensor prototype; (**e**) Preparation of hollow In_2_O_3_ nanofibers, N-GQDs, and PANI/N-GQDs/hollow In_2_O_3_ nanofiber composites-based gas sensor. Reproduced with permission from [67,101,108,115,120]. Copyright 2018, 2021, Royal Society of Chemistry; copyright 2016, 2019, Elsevier; copyright 2021, MDPI.

ZnFe_2_O_4_/GQDs nanocomposites were prepared by hydrothermal method, employing GQDs, Zn(NO_3_)_2_·6H_2_O, Fe(NO_3_)_3_·9H_2_O, and NaOH as the precursors, which favored the interaction with acetone [111]. Zhang et al. [108,109] developed p-p heterostructures between B-GQDs and Ag-LaFeO_3_ through different strategies, respectively realizing the gas sensing of formaldehyde and benzene, in which Ag-LaFeO_3_ was prepared by the sol–gel method combined with microwave chemical treatment. The B-GQDs/Ag-LaFeO_3_ prepared by simple ultrasonic mixing and stirring exhibited excellent performance in detecting low concentration of formaldehyde at low operating temperatures [109]. In addition, in order to achieve a highly selective benzene sensor, the further modification of Ag-LaFeO_3_ was carried out using the molecular imprinting method with benzene as the template, and then B-GQDs/Ag-LaFeO_3_ was synthesized by microwave chemical treatment (Figure 5a) [108].

#### 2.2.5. Metal Phthalocyanine (MPc)/GQDs

MPc had a high saturation response value, but its low conductivity and slow response speed limited its practical application. Jiang et al. [58,101] obtained a series of novel hybrid materials consisting of GQDs and MPc, where GQDs were fixed to the MPc surface by π-π stacking. Cobalt phthalocyanine (CoPc) derivatives containing carboxyl groups (COOH), hexafluoroisopropanol (HFIP), and hexafluorbisphenol A (6FBPA) substituents were prepared for the detection of NO_2_ and dimethyl methylphosphonate (DMMP) gases, and good gas response properties were obtained at room temperature (Figure 5b).

#### 2.2.6. Polymer/GQDs

A highly efficient and simple N-GQDs/PEDOT-PSS sensor system was developed through coating a highly homogenous drop coating on interdigitated Au electrodes on a large area of silicon substrate and used for methanol sensing at room temperature, in which N-GQDs/PEDOT-PSS was prepared under simple sonication and stirring of N-GQDs and PEDOT-PSS [112]. Gavgani et al. [67] also proposed an innovative flexible NH_3_ sensor based on S, N-GQDs/PANI (polyaniline) hybrid loading on PET thin films, which were polymerized through in situ chemical oxidation of S, N-GQDs, aniline, HCl, and ammonium persulfate (APS) (Figure 5c).

#### 2.2.7. Metal Oxide/Polymer/GQDs Composites

Zhang et al. [120] synthesized ZnO/S, N-GQDs/PANI ternary nanocomposites through in situ polymerization of aniline, HCl, S, N-GQDs, APS, ZnO, and NaOH. The ZnO nanopolyhedra was obtained by precipitation of Zn(NO_3_)_2_·6H_2_O and 2-methylimidazole (2-MeIM) using a metal-organic frameworks (ZIF-8) as a template at room temperature (Figure 5d). ZnO/S, N: GQDs/PANI nanocomposites were prepared on a flexible PET substrate with interdigital electrodes, which was a promising material for room temperature acetone sensing. Hollow In_2_O_3_ nanofibers prepared by effective combination of electrospinning and high-temperature calcination were coated with N-GQDs through electrostatic interactions [115]. Using N-GQDs coated hollow In_2_O_3_ nanofibers as the core, PANI/N-GQDs/In_2_O_3_ ternary composites were synthesized by in situ chemical oxidative polymerization (Figure 5e). This ternary composite sensor was highly sensitive at room temperature to detect NH_3_, which was crucial for detecting liver or kidney diseases in human respiration.

#### 2.2.8. Metal Sulfide/GQDs Composites

Yang et al. [122] proposed a novel 3D structured sensor based on MoS_2_/rGO/GQDs hybrids with improved NO_2_ gas sensing performance. MoS_2_ nanoflowers and GQDs nanoparticles were anchored onto rGO nanosheets by hydrothermal method to obtain MoS_2_/rGO/GQDs hybrids. MoS_2_ nanoflowers were prepared by hydrothermal treatment of sodium molybdate dehydrate and thiourea. The incorporation of GQDs inhibited the aggregation of MoS_2_/rGO nanocomposites, significantly improving the uniform distribution of rGO and MoS_2_ nanosheets, and providing many reaction sites for NO_2_ gas adsorption.

#### 2.2.9. GQDs/SiNW

Li et al. [105] proposed a new strategy to manufacture silicon-based gas detectors by using vertically aligned silicon nanowire (SiNW) arrays as skeletons and platforms, and modified with chemically inert GQDs to protect SiNW from oxidation and promote the interaction between charge carriers and analytes. Then, the radial core-shell GQDs/SiNW arrays were assembled into a resistor-based gas detection system, and NO_2_ was used as the model analyte for evaluation. The detection of trace NO_2_ showed ultrahigh sensitivity at room temperature and greatly shortened recovery time, which was of great significance for practical applications. The preparation methods and synthesis parameters were the main factors determining the surface properties of the materials, which in turn determined their gas sensitivity to specific target gases. The next section focuses on the gas sensing mechanisms of GQDs and their composites, and provides the specific principles for the sensing characteristics of each component.

## 3. Gas Sensing Mechanisms

### 3.1. Gas Sensing Mechanism of GQDs

**Figure 6:**
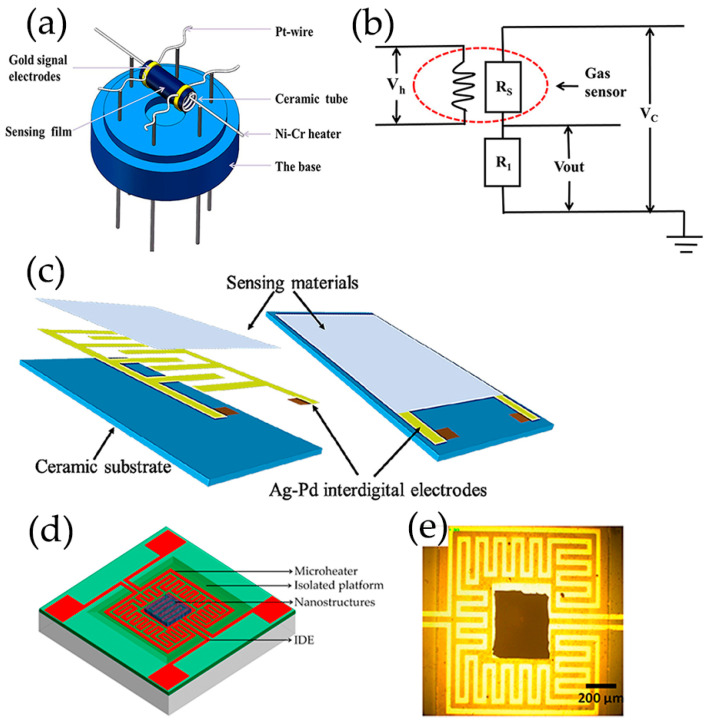
(**a**) Schematic illustration of the structure of a tubular gas sensor; (**b**) Circuit diagram of a tubular gas sensor; (**c**) Schematic illustration of the structure of an IDE gas sensor; (**d**) Schematic diagram; and (**e**) photographic image of a MEMS-based gas sensor. Reproduced with permission from [132,133,134]. Copyright 2020,2016, Elsevier.

This section emphasizes the importance of surface properties of pure GQDs on prompting the better sensing characteristics. The gas sensing mechanism of pure GQDs is usually attributed to adsorption and desorption of sensing species on the surface and edges of GQDs as donors or acceptors, resulting in changes in conductivity of GQDs [68,121]. The physical structures of a chemiresistor sensor are illustrated in Figure 6, where the changes in conductivity of sensing materials occur [131,132,133,134]. A tubular gas sensor consists of a ceramic tube, a Ni-Cr heater, gold signal electrodes, Pt wires, a sensing film, and a base. The circuit diagram of the tubular gas sensor is shown in Figure 6b. The interdigitated electrode (IDE)-based gas sensors consist of a ceramic substrate, interdigital electrodes, and sensing materials (Figure 6c). Micro-Electro-Mechanical System (MEMS)-based gas sensors are constructed on a silicon-wafer platform based on photolithography microelectronics manufacturing and post-processing techniques (Figure 6d,e).

**Figure 7:**
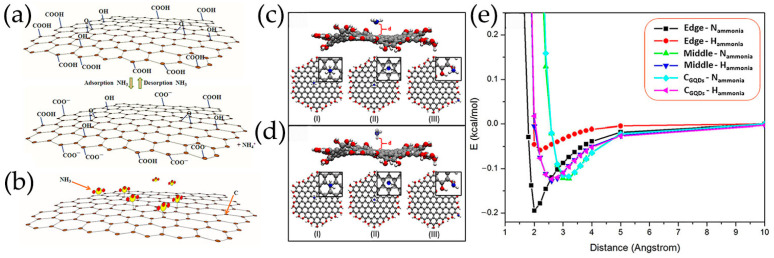
Sensing mechanism of GQDs to NH_3_, (**a**) sensor A, (**b**) sensor B. The interaction model between OH-GQDs and NH_3_ molecules through (**c**) N atoms and (**d**) H atoms of NH_3_ molecule absorbed (**I**) in the middle, (**II**) at the carbon atom and (**III**) at the edge of OH-GQDs, (**e**) The relationship between the interaction energy and the adsorption distance of GQDs-NH_3_ with different adsorption sites and configurations. Reproduced with permission from [68,121]. Copyright 2015, 2020, Elsevier.

The NH_3_ molecules are absorbed into the hollow sites by physical adsorption mechanisms without changing the electronic properties of GQDs. Two gas sensors with opposite current responses were obtained by adjusting the pH values of aqueous GQDs with COOH functional groups at the edges in acidic (sensor A) and neutral (sensor B) (Figure 7a,b) [68]. When acidic sensor A is exposed to NH_3_ gas with a certain humidity, functional COOH tends to ionize to COO^–^ and H^+^, and then H^+^ ions react with NH_3_ gas to form NH_4_^+^. Usually, neutral GQDs are considered to be p-type semiconductors with holes as the main charge carriers. When NH_3_ adsorption occurs, the electron donor NH_3_ on GQDs may cause electrons transfer from NH_3_ to GQDs, resulting in a decrease in the density of holes in GQDs, and thus reducing the conductivity of GQDs, whereas hydroxyl-functionalized GQDs (OH-GQDs) are prepared by hydrothermal treatment of pyrene exhibiting n-type conductivity [121]. Before exposure to NH_3_ gas, the oxygen species are chemically adsorbed on OH-GQDs sensing membrane surface. When exposed to NH_3_ vapor, NH_3_ molecules react with oxygen substances and provide electrons to n-type OH-GQDs. This process leads to an increase in electron carrier density and a decrease in resistance, which has been demonstrated by the calculation of self-consistent charge density functional tight-binding (SCC-DFTB) (Figure 7c–e). The N atoms of NH_3_ exhibit the highest level of interaction at the edge of OH-GQDs, and OH functional groups at the GQDs edge play an important role in the NH_3_ sensing mechanism at room temperature.

Therefore, modifying GQDs through edge functionalization is a promising method for obtaining high-performance gas sensors with high selectivity for the desired target gases. However, to further improve sensing properties, GQDs have been used as a modifier/composite with metals and metal oxides. Therefore, in order to further clarify this point, the gas-sensitive mechanism of GQDs based composites is introduced in the next section (Section 3.2)

### 3.2. Gas Sensing Mechanism of GQDs-Based Composites

**Figure 8:**
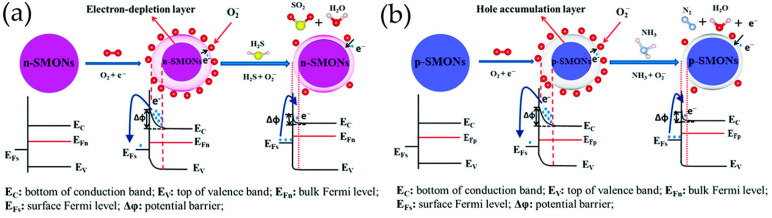
Sensing mechanism of (**a**) n-type SMONs for reducing H_2_S gas and (**b**) p-type SMONs for reducing NH_3_ gas. Reproduced with permission from [15]. Copyright 2019, Royal Society of Chemistry.

Chemiresistive gas sensing is based on the resistance changes resulting from chemical interactions between target gas molecules and oxygen ions adsorbed on semiconductor surfaces [15,17,41]. The degree of change in resistance depends on the type and concentration of the gas. The direct interaction between the sensing layer and the target gas results in changes in the physicochemical properties of the sensing layer [41]. Essentially, when surrounded by air, chemisorbed oxygen molecules on the surface of the sensing layer extract electrons from conduction band of surface layer, thus forming negatively charged oxygen ions in the form of O_2_^−^, O^−^ and O^2−^ at different working temperatures (see Equations (1)–(4)) [15], which deplete the surface layer of n-type materials (a type of semiconductor with most charge carriers being electrons, such as SnO_2_, ZnO, In_2_O_3_, TiO_2_, Fe_2_O_3_, Fe_3_O_4_), or form an accumulated hole layer in p-type materials (a type of semiconductor with most charge carriers being holes, such as LaFeO_3_) [41]. Therefore, the conductivity of the sensing layer decreases (n-type) or increases (p-type), thus resulting in an increased (n-type) or decreased resistance (p-type). When surrounded by a target gas, gas molecules are absorbed on the sensing layer surface and react with these chemically adsorbed oxygen ions. If the target gas is a reducing gas, such as H_2_S, NH_3_, HCHO, or acetone, electrons are released back into the electron depletion layers (n-type) or hole layers (p-type), thereby altering the conductivity of the sensing layer, resulting in a decreased (n-type) or increased resistance (p-type) [15,41]. However, for an oxidizing gas, such as NO_2_, capturing more electrons from sensing layer surface will widen the electron depletion layer (n-type) or hole layer (p-type), resulting in an increase (n-type) or decrease in resistance (p-type) (Figure 8) [15,41].

O_2(gas)_ ↔ O_2(ads)_ (1)

O_2(ads)_ + e^−^ ↔ O_2_^−^_(ads)_ (<100 °C) (2)

O_2_^−^_(ads)_ + e^−^ ↔ 2O^−^_(ads)_ (100–300 °C) (3)

O^−^_(ads)_ + e^−^ ↔ O^2−^_(ads)_ (>300 °C) (4)

Among the reported gas sensors based on GQDs, many reports have shown that modifying GQDs on the surface of metal oxides or polymers can improve gas sensitivity by forming heterojunctions at the interface between metal oxides/polymers and GQDs. GQDs act as an electronic medium at the interface heterojunction and provide a large number of active sites for the composite. Active sites allow chemically adsorbed oxygen to react with target gas molecules, further enhancing the gas sensitivity of the composites [122]. Therefore, for GQDs-based composites, the significant enhancement of gas sensing behaviors can mainly be reflected in the following aspects.

#### 3.2.1. p-n/p-p Heterojunctions

The formation of heterojunctions plays an important role in improving gas sensing performance, which results in the redistribution of charge carriers at the interface, reducing the enthalpy and activation energy required to adsorb target gas molecules [119]. At p-n heterojunctions between p-type and n-type sensing materials, electrons in conduction band of n-type materials will transfer through interface to the lower energy valence band states of p-type materials. Therefore, due to the recombination of electrons and holes, a depletion layer will form at p-n heterojunction [15]. For p-p heterojunctions, the main charge carriers are holes. Due to the different valence band energies of different materials, charge carriers are transferred from one p-type material (with a higher energy valence band state) to another p-type material (with a lower energy valence band state). Therefore, a hole depletion region is formed on the first material surface (with a higher energy valence band state), while a hole accumulation region is formed on the second material surface (with a lower energy valance band state) [15].

**Figure 9:**
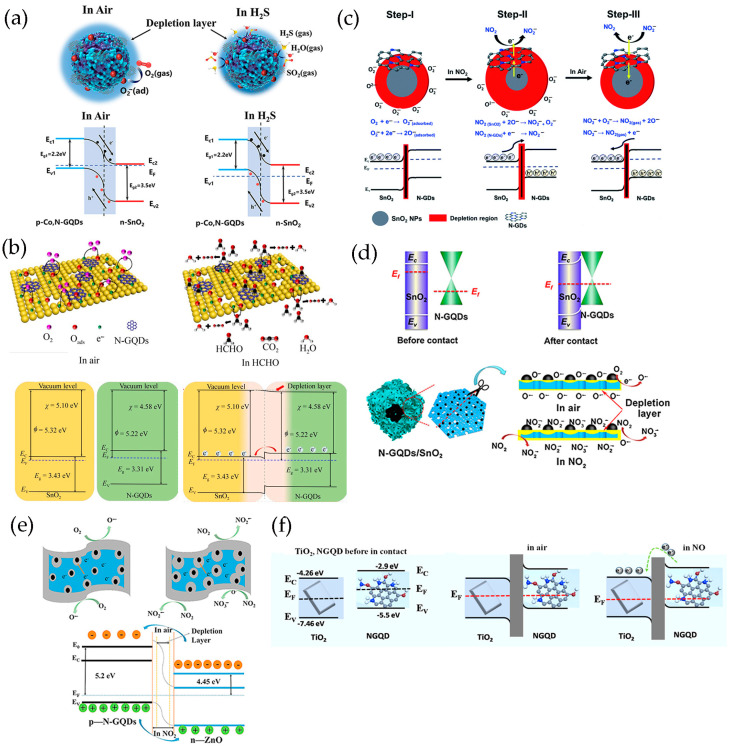
(**a**) H_2_S sensing mechanism and energy band structure of Co, N-GQDs/SnO_2_; (**b**) HCHO sensing mechanism and energy band structure diagram of N-GQDs/SnO_2_; (**c**,**d**) NO_2_ sensing mechanism and energy band structure of N-GQDs/SnO_2_; (**e**) Sensing mechanism of pure ZnO and N-GQDs; (**f**) Energy band structure of TiO_2_/N-GQDs. Reproduced with permission from [100,113,114,116,118]. Copyright 2021, Springer; copyright 2020, Royal Society of Chemistry; copyright 2021, Elsevier; copyright 2020, American Chemical Society.

In general, holes are the main charge carriers in GQDs due to their p-type semiconducting properties. The hole concentration of GQDs can be increased greatly by the addition of Co, and the valence electrons of Co are more beneficial for charge electron transfer [119]. The p-n heterojunction formed between GQDs and metal oxides is very important to improve the sensing performance of GQDs-based composites (Figure 9). To determine the formation of heterojunctions and electron transfer mechanism, the band gap (E_g_) and Fermi level (E_F_) of GQDs and metal oxides are calculated using ultraviolet–visible diffuse reflectance spectroscopy, linear potential scanning, and ultraviolet photoelectron spectroscopy (UPS) [70,116]. When N-GQDs and SnO_2_ come in contact, electrons are transferred from SnO_2_ to N-GQDs until their E_F_ aligns with each other. The construction of N-GQDs/SnO_2_ heterojunction causes the bending of energy band at the interface of heterojunction, forms an additional interface depletion layer, promotes charge carrier separation, and reduces the interfacial charge transfer resistance of composite materials (Figure 9a–d) [135,136]. In addition, Mott–Schottky diagrams of N-GQDs show that doping with N atoms results in the formation of p-n heterojunctions in N-GQDs framework. The p-n interface in N-GQDs can further promote carrier separation and transfer, so that the GQDs sensor surface has a higher potential barrier and generates more oxygen species [70,113]. As a result, the increase of electron concentration in N-GQDs/SnO_2_ is more beneficial to capture electrons for target gas molecules on the material surface, improving the gas-sensitive properties [100,116,118]. The planar π-conjugated structure of GQDs in composite materials can suppress electron–hole pairs recombination, forming a more stable thick space-charge layer and high potential barrier [137]. The interface of GQDs/ZnO nanoparticles belongs to a forward-biased Schottky barrier, which makes electrons easier to be trapped or migrated [57,107,110,138] (Figure 9e). The TiO_2_/N-GQDs sensor performs much better when driven by ultraviolet light, responding 2.6 times better than that of sensors without ultraviolet light. The enhancement in performance under ultraviolet light can be attributed to the efficient production and separation of photoinduced charge carriers, as well as the prevention of electron-hole pairs recombination due to TiO_2_/N-GQDs heterojunction formation (Figure 9f) [113].

**Figure 10:**
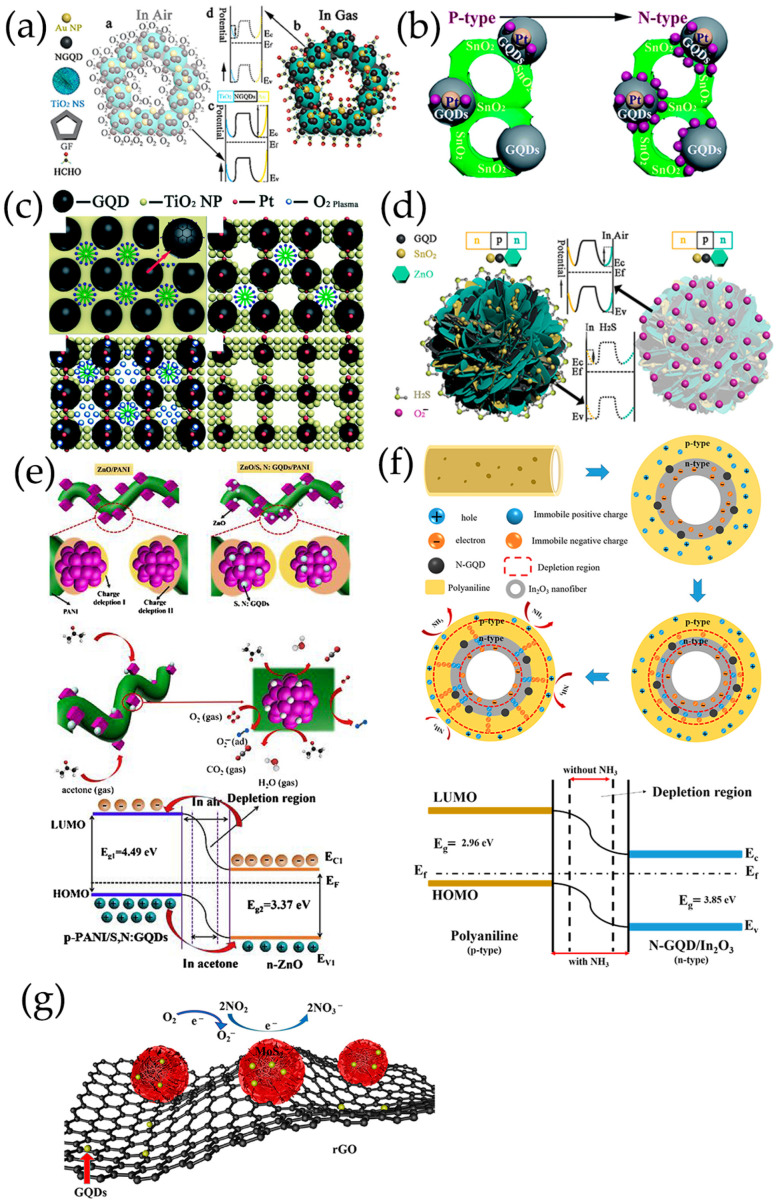
(**a**) HCHO sensing mechanism and energy band structure of N-GQDs/TiO_2_/graphene foam; (**b**) Mechanism of acetone concentration changes in ordered nanoporous 5 wt% GQDs/Pt-SnO_2_ thin films on the transition of p- to n-type acetone sensing behavior; (**c**) Synthetic mechanism of PtO_x_/GQDs/TiO_2_ sensing film; (**d**) Schematic diagram of interface band configuration of GQDs/SnO_2_/ZnO nanostructures under different atmospheres; (**e**) Mechanism diagram of acetone sensing in ZnO/S, N-GQDs/PANI nanocomposite, and p-n heterojunction in ZnO/S, N-GQDs/PANI nanocomposite; (**f**) PANI/GQDs/hollow In_2_O_3_ nanofiber composite sensor sensing mechanism diagram and energy band gap structure diagram; (**g**) Sensing mechanism of MoS_2_/rGO/GQDs composites. Reproduced with permission from [83,98,99,102,115,120,122]. Copyright 2020, Elsevier; copyright 2016, 2017, Royal Society of Chemistry; copyright 2020, American Chemical Society; copyright 2019, Elsevier; copyright 2021, 2022, MDPI.

Adsorbed oxygen has a significant impact on gas detection performance. The thickness of the electron depletion layer is determined by the number of oxygen ions formed by the adsorbed oxygen molecules capturing electrons. Chen et al. [116] proved that the amount of oxygen adsorbed by N-GQDs/SnO_2_ is higher than that of original SnO_2_, confirming that N-GQDs/SnO_2_ surface adsorbed more oxygen ions, which makes the electron depletion layer thicker and may have higher fundamental resistance than original SnO_2_. The interconnected graphene network promotes rapid electron transfer, and N-GQDs also facilitate rapid charge transfer, which increases the affinity between nanoporous TiO_2_ nanospheres and carbon-based scaffolds, and promotes the formation of p-n heterojunctions, thus improving the sensing performance of composite materials [99]. N-GQDs/TiO_2_/graphene foam heterojunctions can provide additional resistance modulation in the oxygen molecular adsorption process by altering the heterojunction potential barrier. As shown in Figure 10a, the carrier concentration on the surface of sensing materials rapidly decreases and the resistance quickly increases. As a result, a thicker electron depletion layer is formed near the surface of TiO_2_ nanospheres. After exposure to HCHO, the surface resistance of N-GQDs/TiO_2_/graphene foam rapidly declines, and the thickness of the electron depletion layer rapidly decreases. Thus, the sensing materials present far greater sensitivity and excellent selectivity to formaldehyde.

In situ preparation of GQDs/Pt modified SnO_2_ thin films with high crystallinization and ordered nanostructure on sensor devices by water vapor hydrothermal method. The thin films exhibit a reversible transition from p-type to n-type sensing and the sensing behavior changes regularly at room temperature as a function of acetone concentration (AC) and GQDs content (GC) (Figure 10b) [98]. Low concentration acetone vapor mainly reacts at p-GQDs/Pt interface layer, which has rich structure defects, affecting the dissociation and adsorption of oxygen as well as the binding of reaction intermediates O_2_^−^ on Pt surfaces. The electrons contributed by the introduction of acetone migrate to the sensing membrane and are confined by p-GQDs/Pt layer, resulting in p-type sensing behavior at room temperature. However, as the acetone concentration increases gradually, the acetone gas molecules cross the heterojunction formed at the interface between GQDs and SnO_2_ nanoparticles. They achieve electron sensitization by adjusting the depletion layer at the heterojunction, thereby reducing the overall resistance of the sensing film. Then, when the subsequent electron concentration exceeds the hole concentration, the conductive type will be reversed from p-type to n-type as the acetone concentration continues to increase. As a result, the sensor is in a low resistance state and exhibits a typical n-type sensing behavior, with S becoming positively charged as the acetone concentration increases.

PtO_x_/GQDs-modified TiO_2_ nanocomposite thin films synthesized in situ on the sensor device are treated by water vapor hydrothermal treatment and oxygen plasma. As a function of oxygen–plasma treatment, the sensing behavior of PtO_x_/GQDs/TiO_2_ on oxygen-functionalized VOCs changes reversibly from p-type to n-type, and the sensing performance of these gas sensors on aromatic VOCs always presents p-type [102]. Under oxygen–plasma treatment for 1 min, because of the deposition of more PtO_x_/GQDs on PtO_x_/GQDs/TiO_2_ surface, isopropanol vapor mainly reacts at the interface layer of PtO_x_/GQDs/TiO_2_. Electrons incorporated by isopropanol migrate to the sensing films and are confined by PtO_x_/GQDs layer, resulting in an inverted layer near the surface, where holes are main carriers and exhibit p-type sensing behavior. After 3 min of oxygen–plasma treatment, PtO_x_/GQDs/TiO_2_ have the largest specific surface area, excellent nanostructure ordering, large pore volume, and highest absorption in the visible region, generating more excited electrons and positive holes. Visible light-induced oxygen ions (O_2_,_hv_^−^) weakly bind to TiO_2_ nanoparticles, exhibiting high reactivity and being responsible for gas sensitivity at room temperature, which is different from chemically adsorbed oxygen ions (O_2,ads_^−^) strongly adhered to TiO_2_ nanoparticle surface. Therefore, oxygen ions (O_2,hv_^−^) induced by visible light play an important role in redox reaction at room temperature. In addition, GQDs improve the response speed by rapidly transferring charge through the interface between GQDs and TiO_2_ nanoparticles. It produces depletion layers instead of inversion layers, and PtO_x_/GQDs/TiO_2_ exhibits n-type sensing behavior. For aromatic VOCs response, benzene preferentially adsorbs on PtO_x_ in the PtO_x_/GQDs layer, as the planar structure of benzene molecules and π-conjugated system strongly adsorb on the GQDs surface. The oxygen-rich functional groups formed on GQDs, particularly carbonyls, not only provide high-energy binding sites for benzene, but also block the direct binding between benzene and PtO_x_/GQDs. In addition, the increase in the functional values of GQDs affected by carbonyl groups leads to a decrease in the Schottky barriers generated at PtO_x_/GQDs heterojunction, resulting in a p-type sensing performance of PtO_x_/GQDs/TiO_2_ with lower response values for benzene (Figure 10c).

In the ternary heterojunctions between p-type GQDs, n-type SnO_2_QNP, and n-type ZnO, GQDs not only come into contact with SnO_2_QNP but also recombine with the ZnO nanosheet (Figure 10d) [83]. Carrier recombination occurs between holes in GQDs and electrons in SnO_2_QNP, and ZnO nanocrystals eventually reach an equilibrium at Fermi level, forming the potential barriers at n-p-n heterojunctions. Therefore, the strong synergistic effect and n-p-n heterojunctions effectively expand the resistance changes caused by oxygen adsorption. The formation of heterojunction between n-type ZnO, p-type PANI and S, N-GQDs at the interface plays a crucial role in improving acetone sensing properties (Figure 10e) [120]. The use of PANI induces the formation of p-n heterojunctions and causes charge carriers to be redistribute at the interface of n-type ZnO, p-type PANI, and S, N-GQDs, reducing the activation energy and enthalpy required to adsorb acetone gas molecules [139]. The existence of S, N-GQDs can form Schottky contacts at the interface of GODs and ZnO, further enhancing sensor response through efficient electrons trapping and migration [140]. In addition, doping of PANI can provide more gas adsorption sites for ZnO/S, N-GQDs/PANI ternary compounds [120]. PANI-coated N-GQDs/In_2_O_3_ nanofibers can generate new chemical bonds on the oxygen-containing defects on the surface of N-GQDs and hollow In_2_O_3_ nanofibers. The p-n heterojunction generated between p-type PANI and n-type N-GQDs/In_2_O_3_ serves as an indicator amplifier, making it easier to detect trace NH_3_ effectively (Figure 10f) [115]. The ternary combination of MoS_2_, GQDs, and rGO can also form p-n junctions at the interface, which is crucial for improving electron transport efficiency and has excellent gas sensitivity to NO_2_ gases (Figure 10g) [122].

**Figure 11:**
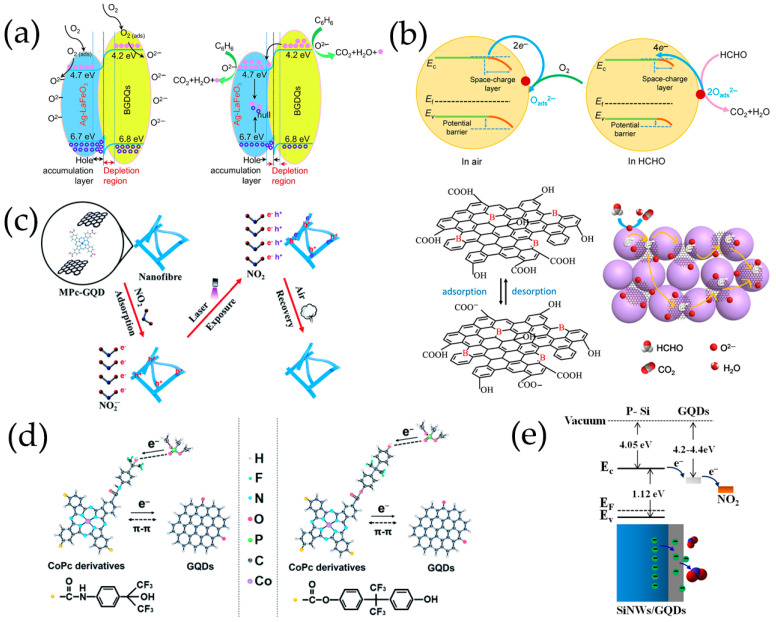
(**a**) Benzene sensing mechanism of B-GQDs/Ag-LaFeO_3_; (**b**) HCHO sensing mechanism of B-GQDs/Ag-LaFeO_3_; (**c**) NO_2_ sensing mechanism of CoPc/GQDs; (**d**) DMMP sensing mechanism of CoPc-HFIP/GQDs and CoPc-6FBPA/GQDs; (**e**) Energy band diagram of GQDs/SiNW heterojunction. Reproduced with permission from [58,101,105,108,109]. Copyright 2018, Royal Society of Chemistry; copyright 2018, Elsevier; copyright 2021, Royal Society of Chemistry; copyright 2017, IOP Publishing.

Zhang et al. [108,109] have prepared B-GQDs/Ag-LaFeO_3_ p-p heterojunction by different synthesis methods, and distinguished benzene and HCHO. The bandgap matching between B-GQDs and Ag-LaFeO_3_ is good, which promotes the separation of electron–hole pairs and improves the carrier transport ability. The resistance of p-p heterojunction depends on the thickness of the hole accumulation layer. Only in the airflow will electrons be captured by adsorbed oxygen and more holes are left on the surface. Once B-GQDs/Ag-LaFeO_3_ are exposed to benzene/HCHO gas, the chemically adsorbed gas depletes the holes near the material surface, reducing the thickness of the hole accumulation layer, leading to a further increase in resistance. Therefore, in the presence of benzene/HCHO vapor, the introduction of B-GQDs reduces/increases the resistance of B-GQDs/Ag-LaFeO_3_. In addition, to achieve a highly selective benzene sensor, Ag-LaFeO_3_ are modified by benzene imprinting, which can specifically recognize benzene during detection (Figure 11a,b) [108]. Phthalocyanine derivatives are p-type semiconductors with low conductivity and low response speed. Jiang et al. [58,101] have prepared novel hybrid materials consisting of GQDs and MPc derivatives, where GQDs are anchored onto the MPc surface through π-π stacking, forming a charge transfer conjugation (Figure 11c). The incorporation of GQDs greatly improves the conductivity of MPc derivatives, resulting in faster response of hybrid materials. NO_2_, as a strong electron acceptor, can capture electrons from CoPc and generate NO_2_^−^ ions. Therefore, abundant holes are generated on the surface of CoPc/GQDs, significantly altering the conductivity of the sensors [58]. DMMP gases are electron donors, resulting in lower hole concentrations of p-type CoPc-HFIP or CoPc-6FBPA. Due to the strong hydrogen bonding between HFIP/6FBPA and DMMP, CoPc-HFIP/GQDs and CoPc-6FBPA/GQDs exhibit excellent response performance to DMMP (Figure 11d) [101]. In addition, the problem of slow MPc recovery is solved by using ultra-low-power laser irradiation. Figure 11e shows the corresponding energy band diagram of GQDs/SiNW heterojunction [105]. The difference between the conduction band location of Si and the work function of GQDs leaves a sufficient internal electric field at GQDs/SiNW interfaces, and electrons are easily extracted from SiNWs and even stored in GQDs layers. Resistance-based GQDs/SiNW array sensors exhibit higher sensitivity, faster recovery, better stability, and repeatability.

#### 3.2.2. Influence of Surface and Intrinsic Sensing Properties of GQDs

Surface functionalization and doping of N, S, and B atoms on GQDs greatly enhance the electrochemical performance of nanocomposites by improving surface area, surface defects, solubility, and the number of surface active sites [141], and regulate the conductivity and electron transfer performance of sensing materials [69,84,111,112,116]. The surface defects and different functional groups of GQDs provide more adsorption sites for gas molecules and enhance the responses of GQDs-based composites [70,98,103,110,116,117].

**Figure 12:**
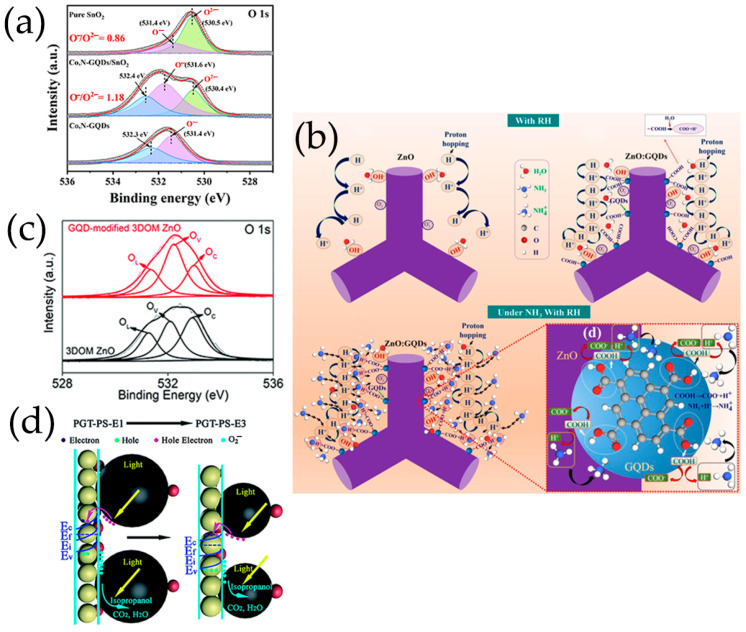
(**a**) O 1s XPS spectra of Co, N-GQDs, pure SnO_2_ and Co, N-GQDs/SnO_2_; (**b**) Schematic diagram of mechanism of ZnO and ZnO/GQDs under relative humidity without and with NH_3_; (**c**) XPS spectra of O 1s of 3DOM ZnO and GQDs/3DOM ZnO; (**d**) VOCs with oxygen functional group sensing mechanism of PGT-PS-E1 and PGT-PS-E3 (PtO_x_/GQDs/TiO_2_ with the oxygen–plasma treated for 1 min or 3 min). Reproduced with permission from [102,106,107,119]. Copyright 2022, Royal Society of Chemistry; copyright 2020, Elsevier; copyright 2019, 2017, Royal Society of Chemistry.

GQDs can form conductive channels, which essentially improve the charge transfer process through PEDOT-PSS or PANI matrices [67,112]. The wide specific surface area of N-GQDs enhances the contact sites with PANI, providing numerous adsorption sites for NH_3_ gas [115]. Co, N-GQDs with carboxyl and hydroxyl groups on the surface can coordinate with SnO_2_ nanospheres, so that Co, N-GQDs are fixed on SnO_2_ nanospheres [119]. Modification of Co, N-GQDs can effectively increase the specific surface area of SnO_2_, thus providing rich reaction sites and accelerating the surface reaction of materials. The small lateral size (~3 nm) of Co, N-GQDs makes it easier for H_2_S gas to reach SnO_2_ materials surface and diffuse [119], improving charge transfer of sensitive materials [117]. Moreover, based on relative peak area ration of chemically adsorbed oxygen to lattice oxygen (O^−^/O^2−^) calculated by the XPS spectrum of O 1s, Co, N-GQDs can provide a large amount of oxygen species to SnO_2_ surface, resulting in the increase of gas reaction sites (Figure 12a) [119,142].

The N-GQDs functionalized SnO_2_ heterostructure prepared by Purbia et al. [118] shows the enhancement of NO_2_ adsorption, as the N-doped GQDs have lower binding energy to NO_2_ than the original GQDs. The density functional theory (DFT) and experimental studies show that N doping or N-H groups are conducive to the adsorption of NO_2_ molecules on N-GQDs surface. The doped high-electron-density N atoms can provide more adsorption active sites for NO_2_ with strong electrophilic ability [70,118,120,143]. Moreover, the higher electronic state density caused by N doping is conducive to electron transfer during NO_2_ sensing [114,144]. Therefore, modifying N-GQDs on SnO_2_ can effectively increase the adsorption of NO_2_, thus enhancing the response of composites and reducing operating temperature [100].

The improvement of NH_3_ sensing performance of ZnO/GQDs sensors is due to the generation of more oxygen-containing groups by the carboxyl and hydroxyl groups of GQDs [106]. As a result, strong hydrogen bonds between oxygen-enriched GQDs and water molecules are generated on their surfaces. This combination is stronger than the van der Waal force and remains unchanged in the whole GQDs, which makes ZnO/GQDs have a higher density of adsorbed water molecules, and results in a high H^+^ molecular density of NH_3_ molecules trapped in air at high relative humidity, thereby improving the response of sensor at room temperature (Figure 12b) [106]. In addition, the number of surface oxygen vacancies during adsorption directly determines the amount of absorbed oxygen ions that can interact with the target gas. The surface oxygen vacancy in 3DOM ZnO modified with GQDs is 40.6%. Because of the functionalization of GQDs, this vacancy is higher than that in 3DOM ZnO samples, thereby improving the sensing performance for acetone (Figure 12c) [107]. With the introduction of GQDs, there are strong synergistic effects between GQDs and 3DOM ZnO, which makes the prepared composites have better conductivity and more oxygen vacancies than 3DOM ZnO [107].

For PtO_x_/GQDs/TiO_2_, GQDs exhibit more oxygen functional groups and smaller size, which facilitates the establishment of more efficient interactions between GQDs and TiO_2_ nanoparticles [102]. This results in a significant reduction in the thickness of the inversion layer between GQDs and TiO_2_ nanoparticles, with the Fermi level higher than the intrinsic level, as shown on the right side of Figure 12d. In addition, the oxygen-rich functional groups in GQDs can advantageously interact well with the oxygen functional groups in VOCs to form electron-rich regions, allowing charge to quickly transfer through the interface between GQDs and TiO_2_ nanoparticles, improving the response speed. In general, S, N-GQDs represent p-type behavior under environmental conditions, due to the electron-withdrawing properties of water absorption, as well as the induction of hole-like carriers by co-doped N and S and oxygen-containing molecules. Thus, exposure of S, N-GQDs to NH_3_ gas as an electron donor leads to a decrease in the number of charge carriers and an increase in resistance [67]. Introducing GQDs into MoS_2_/rGO/GQDs ternary hybrids can prevent the aggregation of MoS_2_/rGO nanocomposites, significantly improve the distribution uniformity of MoS_2_ and rGO nanosheets, and provide numerous reaction sites for chemically adsorbed oxygen reaction with NO_2_, further improving the gas sensitivity of the hybrids [122]. The chemically inert GQDs decorative layer can not only protect SiNWs array from oxidization, but also improve electron transfer across sensor/analyte interfaces, which facilitates the response and recovery process during detection [105].

#### 3.2.3. Morphology of Sensing Materials

The high sensing properties of N-GQDs/SnO_2_ comes from the 2D structure of SnO_2_ nanosheets, which increases the specific surface area of the material and helps to improve the adsorption of HCHO molecules [117]. SnO_2_ nanosheets prepared with GO as the template have a unique ultrathin porous 2D structure, which can reduce the transport pathways, provide more active sites and a large specific surface area, and facilitate the adsorption of HCHO gas molecules, so as to achieve ultra-high response. When the grain size of the sensing material is less than (or equal to) twice the thickness of the depletion layer, all grains on the surface conform to the depletion zone. Therefore, conductivity can be controlled by crystal grains [145]. The average grain size of SnO_2_ (~6 nm) is consistent with the reported thickness of SnO_2_ depletion layer (~3 nm). This indicates that SnO_2_ grains in the ultrathin nanosheets are exhausted [145], which may mean that most SnO_2_ are active and provide rich active sites for oxygen species and HCHO molecules.

**Figure 13:**
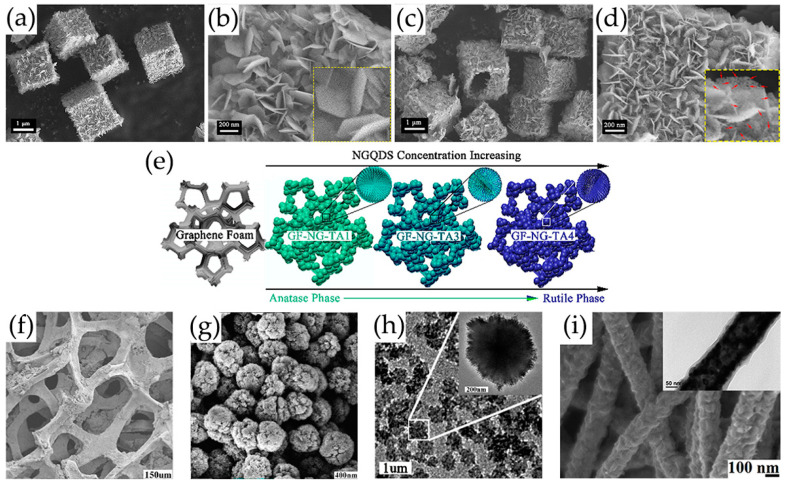
SEM images of (**a**,**b**) SnS_2_ and (**c**,**d**) N-GQDs/SnO_2_; (**e**) Fabrication for N-GQDs/TiO_2_/graphene foam; FESEM micrographs of (**f**) N-GQDs/TiO_2_/graphene foam and (**g**) hierarchical TiO_2_ nanospheres; (**h**) TEM images of N-GQDs/TiO_2_/graphene foam; (**i**) SEM and TEM images of 20 wt% PANI/hollow In_2_O_3_ nanofiber. Reproduced with permission from [99,100,115]. Copyright 2021, 2020, Elsevier; copyright 2021, MDPI.

Lv et al. [100] (Figure 13a–d) prepared a novel ultrathin mesoporous nanosheet SnO_2_ hierarchical hollow cube through a simple sulfidation–oxidation method, which provides beneficial adsorption and diffusion pathways and can quickly adsorb and desorb NO_2_. Moreover, the rich mesoporous and nanocrystalline boundaries provide more depletion layers for the sensing process, thus increasing the resistance changes after exposure to NO_2_ gas. The obtained nanoporous TiO_2_ based on graphene network is subsequently functionalization with N-GQDs, which has a high specific surface area, provides rich reaction sites for gas molecule diffusion, and improves the response capability of gas sensors [99]. As the concentration of N-GQDs increases, the crystalline phase of TiO_2_ gradually transforms from anatase phase to rutile phase, as shown in Figure 13e–i. The co-contribution of TiO_2_ nanospheres with 2 wt% N-GQDs results in a low surface free energy, which lays the foundation for the high surface area of the obtained N-GQDs/TiO_2_/graphene foam sensing materials, showing a high response to 6.1–40 ppb HCHO at 150 °C [99]. In addition, the high proportion of (001) surface in N-GQDs/TiO_2_/graphene foam plays an important role in improving its gas-sensitive performance and maintaining its stability. The ZnO porous nanostructure modified by S, N-GQDs, and PANI nanocapsules provides a large number of reaction sites and allows gas molecules to diffuse inside and outside the membrane layers. More oxygen molecules are adsorbed on ZnO surface and react with acetone, resulting in significant changes in resistance [120]. The wide specific surface area of hollow In_2_O_3_ nanofibers enhances the contact sites with PANI and provides a considerable number of adsorption sites for NH_3_ gas (Figure 13e) [115].

**Figure 14:**
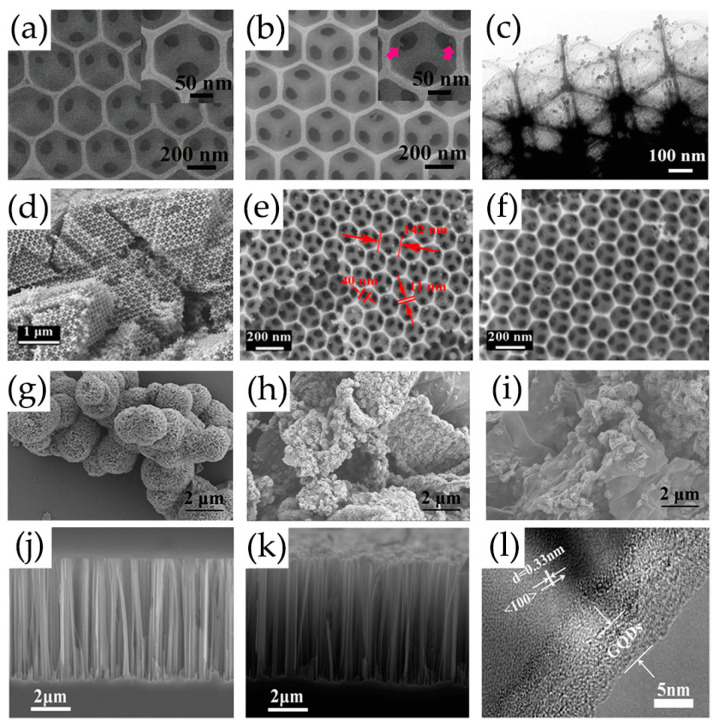
SEM images of (**a**) 3DOM ZnO and (**b**) GQDs/3DOM ZnO, (**c**) TEM image of GQDs/3DOM ZnO). SEM images of (**d**,**e**) 3DOM In_2_O_3_ under different magnifications, and (**f**) N-GQDs/In_2_O_3_. SEM images of (**g**) MoS_2_, (**h**) MoS_2_/rGO and (**i**) MoS_2_/rGO/GQDs. Cross-sectional SEM image of (**j**) SiNW array before surface modification and (**k**) GQD/SiNW; (**l**) TEM image of a single SiNW enveloped by GQDs layer. Reproduced with permission from [70,105,107,122]. Copyright 2019, Royal Society of Chemistry; copyright 2020, American Chemical Society; copyright 2022, MDPI; copyright 2017, IOP Publishing.

The results of nitrogen adsorption isotherm and Barrett–Joyner–Halenda (BJH) pore size distribution show that 3DOM structure modified by GQDs can maintain a large specific surface area and hierarchical pore distribution, and have high gas accessibility and easily quickly approach and analyze gas molecules, making the sensors more active in gas detection (Figure 14a–f) [70,107]. The alternating stacking 3D nanostructures based on MoS_2_/rGO/GQDs ternary hybrids provide numerous good adsorption sites for NO_2_ gas, which are conducive to electron transport and further improve the gas sensitivity of MoS_2_/rGO/GQDs [122]. The rGO nanosheets are used as carrier transport channels and substrates for growing MoS_2_ nanoflowers (Figure 14g–i) [122]. A novel gas phase ultrasensitive detection structure based on GQDs-modified SiNW arrays has been proposed, in which vertically arranged SiNW arrays are used as skeletons and platforms because of their large surface areas and sufficient gas molecule diffusion gaps (Figure 14j–l) [105].

#### 3.2.4. Doping/De-Doping Process of Polymers

Reversible acid-base doping/de-doping processes are widely used to explain the conductivity changes of conductive polymers for acidic/alkaline analytes [67,112]. When methanol molecules are physically adsorbed on N-GQDs/PEDOT-PSS surface, the holes on the conductive N-GQDs/PEDOT-PSS will interact with methanol, which gives electrons. This not only leads to the increase in the delocalization of conjugated π-electrons in the sensing film, but also leads to the decrease of charge carriers and conductivity of the sensing film [112,146]. When S, N-GQDs/PANI are exposed to NH_3_, not only does the de-doping process occur, but also PANI nanoparticles can gradually change from a conductive state to an insulating state, resulting in an increased resistance [67].

#### 3.2.5. Swelling Process of Polymers

The swelling process of analytes diffusing into conductive polymer systems is a widely observed phenomenon in VOCs detection. N-GQDs embedded in PEDOT-PSS act as conductive pathways that facilitate electronic hopping. The swelling process can lead to the separation of N-GQDs and destroy the conductive pathways in N-GQDs/PEDOT-PSS. The increase in the distance between PEDOT-PSS and the decrease in the conductive path of N-GQDs result in a significant increase in the resistance of N-GQDs/PEDOT-PSS exposed to methanol, thereby enhancing the methanol response [112]. Similarly, when NH_3_ molecules diffuse into S, N-GQDs/PANI, the swelling process can also disrupt the connectivity of the conductive pathways of S, N-GQDs, resulting in a significant increase in the resistance of S, N-GQDs/PANI sensors when exposed to NH_3_ [67]. Therefore, due to the observed high response and selectivity towards polar molecules, the direct charge transfer process is most likely the primary process for methanol or NH_3_ sensing in the sensors.

## 4. Gas Sensing Performance of GQDs-Based Composites

The parameters for evaluating gas sensing characteristics include gas response, selectivity, operating temperature, response/recovery time, stability, and detection limit (LOD) [23]. These parameters are mainly determined by three factors, namely receptor function, transducer function and utility, corresponding respectively to the ability to interact with the target gas, convert the surface chemical interaction into an electrical signal, and the ability to approach the target gas [147]. The response value can be defined in two different ways: (i) the ratio of the total change to the original value of the measured parameter, in which case it is usually reported as a percentage ∆*R*/*R_a_*, or (ii) the ratio of baseline to peak magnitude in n-type *R_a_*/*R_g_* and p-type *R_g_*/*R_a_* materials, and vice versa, where *R_a_* and *R_g_* are resistances of sensors in air and target gas, respectively [41]. Response or recovery time is defined as the time it takes for the sensor to reach 90% or 10% of the final response from injecting or extracting the target gas, respectively [15]. Table 3 highlights the benefits of all available chemiresistive gas sensors on GQDs-based composites. Readers can refer to the original report for the complete sensing analysis.

### 4.1. Formaldehyde

**Figure 15:**
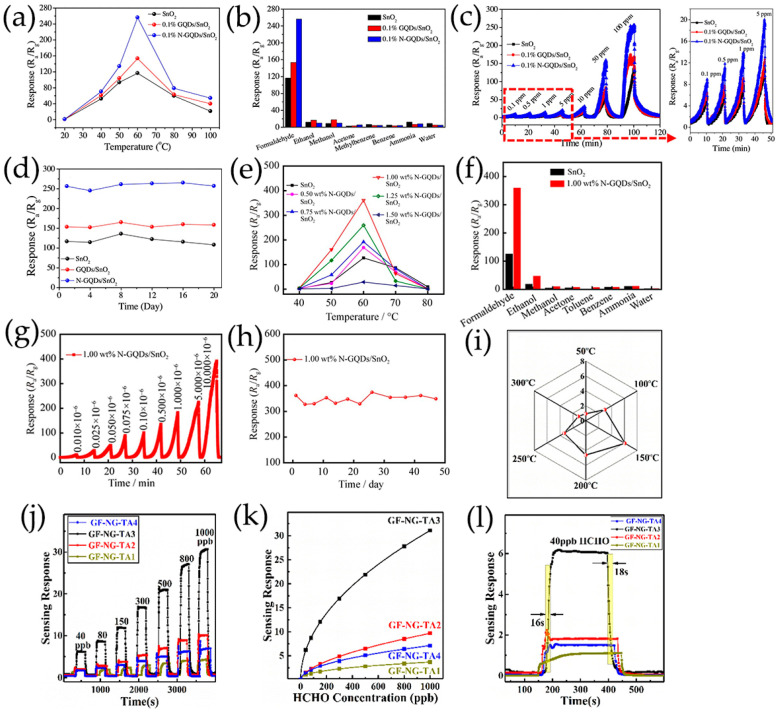
Response of SnO_2_, GQDs/SnO_2_, and N-GQDs/SnO_2_ to (**a**) 100 ppm HCHO with the temperature range of 20–100 °C and (**b**) 100 ppm different gases at 60 °C; (**c**) Response/recovery curves to 0.1–100 ppm HCHO and sensor response to 0.1–5 ppm HCHO; (**d**) Long-term stability to 100 ppm HCHO at 60 °C; (**e**) Sensor responses of N-GQDs/SnO_2_ to 10 ppm HCHO with the temperature range of 40–80 °C; (**f**) Selectivity of SnO_2_ and N-GQDs/SnO_2_ to 10 ppm different gases at 60 °C; (**g**) Dynamic response curves of N-GQDs/SnO_2_ sensors to HCHO orderly with increasing concentration at 60 °C; (**h**) Long-term stability of N-GQDs/SnO_2_ to 10 ppm HCHO at 60 °C. (**i**) Radar plot and gas response of GF-NG-TA3 to 40 ppb HCHO at various working temperature; (**j**) Response curves of GF-NG-TA1~GF-NG-TA4 determined at different concentrations of HCHO gas; (**k**) Functional relationship between normalized responses of GFNG-TA1~GF-NG-TA4 and HCHO gas concentrations; (**l**) Response curves of GF-NG-TA1 to GF-NG-TA4 determined at 40 ppb HCHO gas; (GF-NG-TA1~GF-NG-TA4 with 0.5, 1, 2 and 4 wt% GQDs). Reproduced with permission from [99,116,117]. Copyright 2019, Elsevier; copyright 2021, Springer; copyright 2020, Elsevier.

Formaldehyde (HCHO) is a well-known indoor air pollutant that is toxic to human beings, and can cause sneezing, coughing, and nausea. It can also undergo intramolecular and intermolecular crosslinks between proteins and DNA, leading to cancer [148,149]. Therefore, the development of HCHO gas sensors to protect people’s health is imperative. The following describes the existing HCHO sensors developed using GQDs and its composite materials. Chen et al. [116,117] synthesized N-GQDs/SnO_2_ for detecting HCHO, in which SnO_2_ obtained by hydrothermal treatment and calcination have the morphology of nanosheets and 2D mesoporous ultrathin structure, respectively. When SnO_2_ was combined with N-GQDs, the gas sensitivity of HCHO was improved more significantly than that of GQDs. The influence of N-GQDs loading amount on final sensing properties was studied. The response value of 0.1 wt% N-GQDs/SnO_2_ nanosheets to 100 ppm HCHO at 60 °C was 256, approximately 2.2 times that of the original SnO_2_ nanosheets (Figure 15a–d) [117]. When adding 1.00 wt% N-GQDs, the detection response of SnO_2_ with 2D mesoporous ultrathin structure to 10 ppm HCHO increased from 120 to 361 at 60 °C [116]. Moreover, the response/recovery curve almost restored its original behavior, indicating that the sensor had sufficient stability and repeatability [116,117]. After 20 and 50 days, the sensitivity of the materials remained relatively at the original value, indicating that the sensor had good long-term stability (Figure 15e–h) [116,117]. The materials also showed good selectivity to HCHO. The response/recovery time of 1.00 wt% N-GQDs/SnO_2_ exposed at 10 ppm HCHO were 330 s and 30 s, respectively. The LOD of 1.00 wt% N-GQDs/SnO_2_ was as low as 0.01 ppm, which was conducive to indoor HCHO detection at 0.08 ppm. The N-GQDs/SnO_2_ sensors had unique sensing properties, mainly due to the high specific surface area of 2D mesoporous nanostructure and the synergistic effects between N-GQDs and SnO_2_. The addition of N-GQDs increased the number of adsorption sites on nanocomposite surface and regulated the conductivity and electron transfer performance of the material.

P-type Ag-LaFeO_3_ (AL) is a promising gas-sensitive material with large specific surface area, abundant reactive oxygen lattice, good thermal stability, controllable structure, and strong reducibility, but its working temperature is high [109]. B-GQDs with p-type semiconductor property could produce a synergistic effect with AL, significantly reducing the working temperature of AL from 90 °C to 55 °C. The response/recovery time of 1 ppm HCHO is 23/30 s. In a novel 3D-structured gas microsensor, gold-modified N-GQDs/TiO_2_ were uniformly and firmly distributed over the whole surface of graphene foam frameworks with high specific surface area, providing rich reaction sites for gas molecules diffusion, and improving the response ability of the gas sensor [99]. The interconnected graphene network promoted rapid electron transfer, while N-GQDs also promoted rapid charge transfer, increasing the affinity between TiO_2_ and carbon-based frameworks, and promoting the formation of p-n heterostructures, thus enhancing the sensing properties of N-GQDs/TiO_2_/graphene foam. N-GQDs/TiO_2_/graphene foam with the highest percentage of (001) facets showed the highest sensitivity to HCHO at ppb-level under high relative humidity, and 2 wt% N-GQDs showed a high response to 6.1–40 ppb of HCHO at 150 °C (Figure 15i–l). The experimental results indicated that almost all oxygen species absorbed on TiO_2_ nanospheres were O^2−^, which had greater activity and energy than O_2_, O_2_^−^, and O^−^, explaining the high response of the as-prepared TiO_2_ nanoparticles. The chemically adsorbed oxygen species and possible reactions with HCHO could be described as Equation (5). The effect of relative humidity on sensing performance of HCHO was evaluated under conditions of 50%, 70%, and 90%RH, indicating the sensing performance decreased when the relative humidity increased from 50% to 90%. More importantly, N-GQDs/TiO_2_/graphene foam also showed excellent selectivity and long-term stability. The number of oxygen vacancies, adsorbed oxygen, and nano-heterojunctions were important factors to improve the sensing performance of nanocomposites for HCHO.

HCHO_(ads)_ + 2O^2−^_(ads)_ → CO_2 (gas)_ + H_2_O _(gas)_ (5)

### 4.2. Acetone

Acetone is an important industrial solvent, which is flammable, explosive, and harmful to human health [14,103,111], but it can serve as a good marker for noninvasive diagnosis of diabetes. According to clinical analysis, the acetone concentration in exhaled gas of diabetic patients is higher than 1.8 ppm [150]. Facing the extremely low target gas concentration and complex background environment, developing a high-performance gas sensor for acetone detection is greatly significant in environmental monitoring and noninvasive diagnosis of diabetes. Acetone sensors reported using GQDs nanocomposites are discussed in this section.

**Figure 16:**
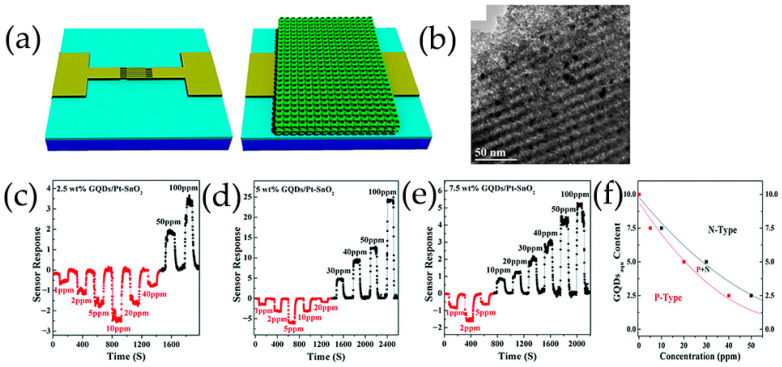
(**a**) Device structure and sensor device with GQDs/Pt-SnO_2_ thin film; (**b**) TEM image of 5 wt% GQDs/Pt-SnO_2_ thin film annealed at 300 °C; Response curves of (**c**) 2.5 wt%, (**d**) 5 wt%, and (**e**) 7.5 wt% GQDs/Pt-SnO_2_ membrane sensors determined at different acetone gas concentrations; (**f**) Transition diagram of GQDs content (GC) and acetone concentration (AC); Reproduced with permission from [98]. Copyright 2016, Royal Society of Chemistry.

In situ preparation of GQDs/Pt-modified nanoporous SnO_2_ thin films with high crystallinity and nanostructural order was carried out by water vapor hydrothermal treatment on sensor devices (Figure 16a,b), which demonstrated the reversible conversion from p-type to n-type acetone sensing at room temperature, and the regular changes in sensing behavior regulated by acetone concentration (AC) and GQDs concentration (GC) [98]. The sensing performance of GQDs/Pt-SnO_2_ thin film sensors was studied by exposing it to acetone gas in the concentration range of 1–100 ppm (Figure 16c–f). After adding GQDs to SnO_2_ sensing film, the sensor response to acetone gas was significantly improved, with a 50% reduction in response time. The SnO_2_ thin film with 2.5 wt% GQDs showed abnormal p-sensing behavior with S < 0, while the SnO_2_ thin film with ≥7.5 wt% GQDs showed a typical n-sensing behavior with S > 0. The sensing response of the sensing film with 5 wt% GQDs was higher than that of other samples. The response/recovery time of GQDs/Pt-SnO_2_ to acetone gas was 5–10 s and 10–15 s, respectively, and decreased with the increase of acetone concentration. The sensing film had a detection limit as low as 1 ppm acetone, which has excellent sensitivity and selectivity. Therefore, binary (AC-GC) transition diagram could be used to design multifunctional sensor systems and control p-n transitions of novel gas-sensitive materials. SnO_2_/GQDs nanocomposites were prepared by solvothermal method, and GQDs content had a significant effect on the response of SnO_2_/GQDs to acetone [103]. The incorporation of GQDs greatly enhanced the response of SnO_2_/GQDs composites, but excessive GQDs content in SnO_2_/GQDs reduced the response of sensor devices. This was mainly because the conductivity of the composites was greatly increased, resulting in a decrease in the ratio of resistance changes detected by the sensor equipment in gas and air to the resistance in air. The sensor based on SnO_2_/GQDs composites showed excellent response and selectivity to acetone vapor at 275 °C. The sensor response to 1000 and 0.1 ppm acetone reached 120.6 and 1.3, respectively. The response/recovery time to 1000 ppm acetone were 17 and 13 s, respectively. ZnFe_2_O_4_/GQDs nanocomposites were prepared by hydrothermal method and showed excellent response and selectivity to acetone at room temperature [111]. The response of ZnFe_2_O_4_/GQDs to acetone at 1000 ppm and 5 ppm was 13.3 and 1.2, respectively. The response/recovery times for 5–1000 ppm acetone were both less than 12 s. When the sensor was exposed to acetone, the oxygen species adsorbed on the surface reacted with acetone, the reaction equations were as follows (Equations (6) and (7)):

CH_3_COCH_3(ads)_ + 8O^−^_(ads)_ → 3CO_2 (gas)_ + 3H_2_O _(gas)_ + 8e^−^ (6)

CH_3_COCH_3(ads)_ + 8O_2_^−^_(ads)_ → 3CO_2 (gas)_ + 3H_2_O _(gas)_ + 16e^−^ (7)

**Figure 17:**
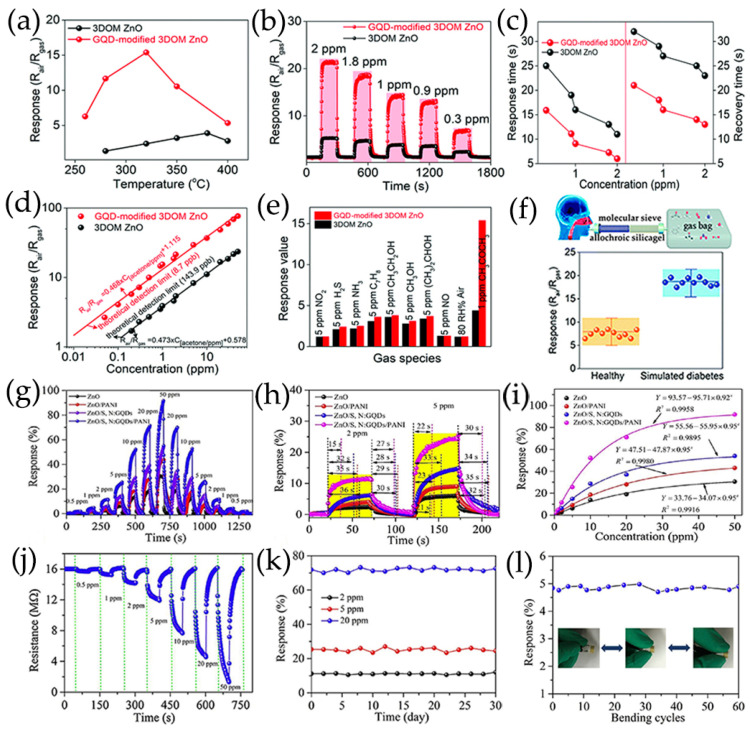
(**a**) Relationship between response and operating temperature of GQD/3DOM ZnO to 1 ppm acetone; (**b**) Dynamic response curve with concentration range of 0.3–2 ppm; (**c**) Response and recovery time to acetone; (**d**) Linear relationship between response and acetone concentrations; (**e**) Selectivity of 3DOM ZnO and GQDs/3DOM ZnO; (**f**) Schematic diagram of respiratory acquisition process and response of GQDs/3DOM ZnO to healthy and simulated diabetic exhaled breath. (**g**) Response of ZnO/S, N-GQDs/PANI to acetone at 25 °C; (**h**) Dynamic response–recovery curves; (**i**) Sensor response as a function of gas concentration; (**j**) Real-time resistance measurement of acetone gas using ZnO/S, N-GQDs/PANI; (**k**) Long term stability of ZnO/S, N-GQDs/PANI for 2, 5, and 20 ppm acetone; (**l**) Gas sensing response of ZnO/S, N-GQDs/PANI to 1 ppm acetone after some bending cycles. Reproduced with permission from [107,120]. Copyright 2019, Royal Society of Chemistry; copyright 2019, Elsevier.

Liu et al. [107] successfully synthesized GQDs/3DOM ZnO nanomaterials by a simple self-assembly template method and spin coating, and carefully studied the gas sensitive characteristics, comparing with the original 3DOM ZnO. With the incorporation of GQDs, the strong synergistic effect between GQDs and 3DOM ZnO and p-n heterojunction made GQDs/3DOM ZnO exhibit more oxygen vacancies and better conductivity than 3DOM ZnO. GQDs/3DOM ZnO had an ordered macroporous structure with a hierarchical aperture (macroscopic size of 286 nm and mesoscale size of 26 nm) and high surface area (88.2 m^2^g^−1^), ensuring high gas accessibility and rapid carrier transport. The results showed that GQDs/3DOM ZnO sensor had very high response to acetone gas (*R_a_*/*R_g_* = 15.2 at 1 ppm), fast response/recovery time (9/16 s), very low theoretical detection limit (8.7 ppb), good selectivity to acetone, and was not interfered with by other gases (Figure 17a–f). Moreover, a GQDs/3DOM ZnO sensor could also accurately distinguish acetone biomarkers in healthy and simulated diabetes samples. These results proved the potential viability of GQDs/3DOM ZnO as a promising high-performance noninvasive real-time diagnosis sensing material for diabetes. ZnO/S, N-GQDs/PANI nanocomposites derived from MOFs were synthesized using a simple one-step in situ polymerization technology and prepared on flexible PET substrates with interdigital electrodes [120]. The sensing performance of ZnO/S, N-GQDs/PANI sensors to acetone was studied by exposure to acetone with different concentrations at room temperature, indicating that ZnO/S, N-GQDs/PANI had high response (11.56 at 2 ppm, 25.6 at 5 ppm), fast response/recovery time (15/27 s to 2 ppm, 22/30 s to 5 ppm), stable repeatability, ppb-level sensitivity (LOD = 0.1 ppm), excellent selectivity, and long-term stability (Figure 17g–l). Therefore, ZnO/S, N-GQDs/PANI film sensor was a suitable choice for ppb-level acetone sensing at room temperature, because ZnO/S, N-GQDs/PANI had a synergistic effect and heterojunction.

### 4.3. Ammonia

Ammonia (NH_3_), as a colorless toxic gas, has been widely used in various industries. However, when it exceeds the 25 ppm threshold in the air, it can harm human skin, eyes, and respiratory system [151]. Detecting NH_3_ in human respiration can serve as a biomarker for diagnosing diseases and exploring physical obstacles in various organs [152]. Therefore, selective detection of NH_3_ is crucial, and the following focuses on sensors using GQDs composite materials.

**Figure 18:**
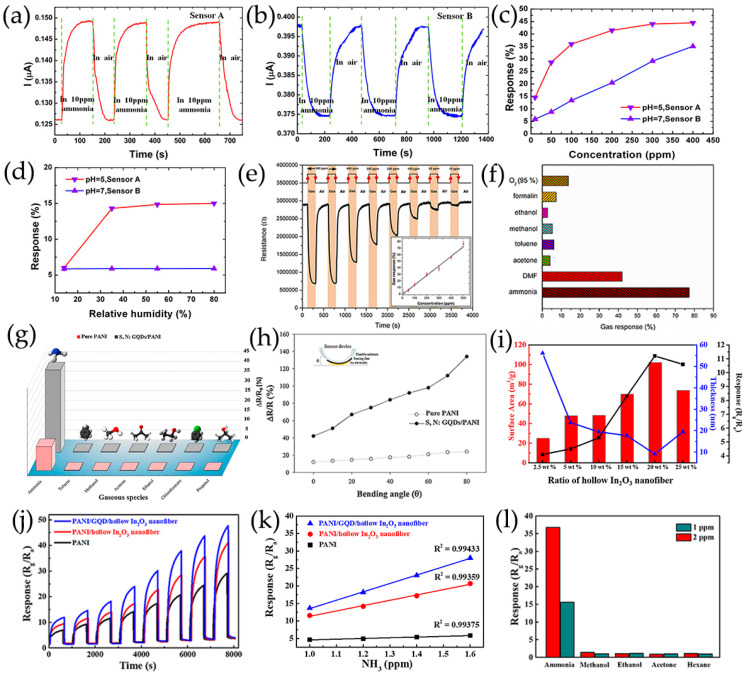
Response of (**a**) sensor A and (**b**) B to 10 ppm NH_3_ for three cycles; Absolute response of sensors A and B (**c**) at various concentrations of NH_3_, and (**d**) to 10 ppm NH_3_ at various relative humidity. (**e**) Resistance changes of OH-GQDs to different concentrations of NH_3_ vapor at room temperature; (**f**) Selective histogram of OH-GQDs to various gases at room temperature. (**g**) Selectivity of flexible pure PANI and S, N-GQDs/PANI to various VOCs vapors of 100 ppm at 25 °C in 57% RH; (**h**) Influence of flexibility of PANI and S, N-GQDs/PANI on the response of 100 ppm NH_3_ at 25 °C in 57% RH. (**i**) Surface area, thickness, and response of PANI/In_2_O_3_ to the loading of In_2_O_3_; (**j**) Dynamic response-recovery curves and (**k**) response-concentration fitting curves of PANI/GQDs/In_2_O_3_; (**l**) Selectivity of PANI/GQDs/In_2_O_3_. Reproduced with permission from [67,68,115,121]. Copyright 2015, 2020, 2016, Elsevier; copyright 2021, MDPI.

Based on acidic and neutral GQDs, a simple solution-based spraying method was demonstrated to manufacture two different NH_3_ sensors with opposite current responses. This method was introduced to improve the device response [68]. When exposed to NH_3_ at room temperature, the manufactured gas sensors showed promising response and selectivity (Figure 18a–d). The acidic GQDs-based gas sensor (sensor A) had an estimated response of −14.9%, with a 90% response/recovery time of 26/21 s. The neutral GQDs based gas sensor (sensor B) had an estimated response of 5.9% and a 90% response/recovery speed of 27/72 s. The response of acidic GQDs sensor to NH_3_ might be due to the deprotonation of carboxyl groups, while the sensing activity of neutral GQDs might be due to electron transfer from NH_3_ molecules to GQDs, resulting in changes in the density of holes in GQDs. OH-GQDs were synthesized by the bottom-up method, and NH_3_ gas sensors were fabricated at room temperature (26 ± 2 °C) with 57 ± 2% RH using pyrene hydrothermal treatment and drip onto an interdigitated nickel electrode [121]. The gas responses of OH-GQDs sensors to NH_3_ with concentrations of 500, 400, 300, 200, 100, 50, and 10 ppm were calculated as 76.63, 55.78, 38.98, 30.00, 14.16, 5.77, and 1.54%, respectively (Figure 18e). The calculated response/recovery time of OH-GQDs sensor under 500 ppm NH_3_ were 64/69 s, respectively. At room temperature, the OH-GQDs sensor exhibited high selectivity against CH_2_O, C_2_H_6_O, CH_3_OH, C_7_H_8_, C_3_H_6_O, and DMF (Figure 18f). The results showed that the OH functional groups on the edge of GQDs played an important role in the sensing mechanism of NH_3_ at room temperature, because the N atom of NH_3_ molecules interacted most with the OH functional groups (Equation (8)). Thus, quantum confinement, edge effects, and the existence of functional groups on GQDs played a vital role in achieving excellent gas sensitive properties.

4NH_3(gas)_ + 5O_2_^−^_(ads)_ → 4NO _(gas)_ + 6H_2_O _(gas)_ + 5e^−^ (8)

A highly selective NH_3_ sensor was successfully proposed based on ZnO and different amounts of GQDs [106]. The NH_3_ sensing results indicated that when NH_3_ concentration was 1000 ppm and GQDs content was 15 μL, the ZnO sensor had the best response with a value of 6047, which was mainly due to carboxyl and hydroxyl groups of GQDs. These oxygen-containing groups enabled the sensor to adsorb water molecules with high density, resulting in the generation of H^+^ molecules to capture NH_3_ in the air at relative humidity. This contributed to the sensor’s high responsiveness and selectiveness to NH_3_ at room temperature (Equations (9)–(12)).

2H_2_O_(l)_ → H_3_O^+^ + OH^−^ (9)

H_3_O^+^ → 2H_2_O_(l)_ + H^+^ (10)

NH_3 (gas)_ + H^+^ → NH_4_^+^ (11)

4NH_3(gas)_ + 3O_2_^−^_(ads)_ → 2N_2(gas)_ + 6H_2_O_(gas)_ + 3e^−^ (12)

A novel flexible NH_3_ sensor was obtained by loading S, N-GQDs/PANI hybrid sensor on PET thin film through chemical oxidative polymerization. It had excellent response (42.3 and 385 at 100 ppm and 1000 ppm, respectively), good selectivity, fast response/recovery time (115 and 44 s, respectively) at room temperature, as well as flexibility, low cost, and wearability (Figure 18g) [67]. Experimental results showed that when NH_3_ concentration was 100 ppm, the response of S, N-GQDs/PANI was about 5 times higher than that of pure PANI. For flexible pure PANI and S, N-GQDs/PANI, the NH_3_ detection limits at 25 °C and 57% RH were 1 ppm and 500 ppb, respectively. In addition, the response of flexible S, N-GQDs/PANI significantly increased with increasing bending angle (Figure 18h). The improvement in gas sensitivity could be attributed to the synergistic effect between S, N-GQDs, and PANI. Acid-base doping/de-doping process, carrier mobility, and swelling process were the possible sensing mechanisms for flexible S, N-GQDs/PANI.

A novel ternary nanocomposite was prepared using conductive polymer PANI, hollow In_2_O_3_ nanofiber, and N-GQDs as electrode materials as gas sensors for detecting NH_3_ in human respiration ranging from 1.0 to 1.6 ppm (Figure 18i–l) [115]. The response value of PANI/N-GQDs/In_2_O_3_ sensor loaded with 20 wt% N-GQDs/In_2_O_3_ for 1 ppm NH_3_ was 15.6, which was about 4.4 times that of PANI sensor. PANI/N-GQDs/In_2_O_3_ sensor was proven to be very sensitive in detecting NH_3_ with the concentration of 0.6–2.0 ppm at room temperature, which was very important for detecting liver or kidney diseases in human respiration. This ternary composite sensor also exhibited greater selectivity and repeatability when exposed to 1.0 and 2.0 ppm NH_3_ at room temperature.

### 4.4. H_2_S

Hydrogen sulfide (H_2_S) is a common toxic, flammable, colorless, and smelly egg gas that can affect the nervous system and poison various metabolic systems of the human body [153]. The threshold for H_2_S is limited to less than 10 ppm according to the safety standards published by OSHA [154]. In addition, H_2_S as a biomarker in exhalation is associated with halitosis. The H_2_S gas content exhaled by halitosis patients exceeds 0.1 ppm [155]. Sensitive real-time monitoring of trace H_2_S (100–500 ppb) in exhaled gas can provide useful information for clinical disease diagnosis [156]. Thus, it is necessary to develop an efficient H_2_S gas sensor. This section introduces the H_2_S sensors reported using GQDs based composites.

**Figure 19:**
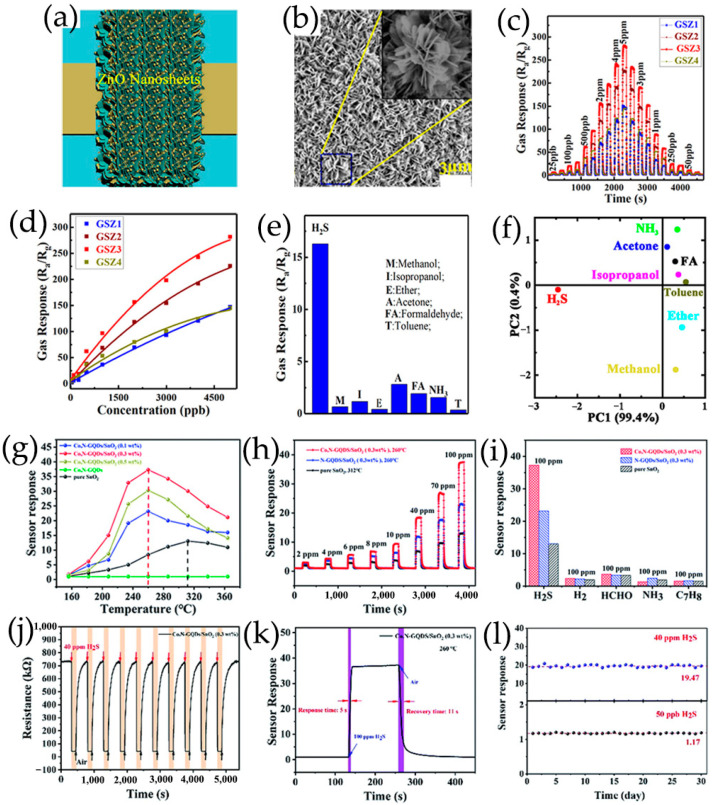
(**a**) Schematic diagram of GQDs/SnO_2_/ZnO; (**b**) SEM images of GQDs/SnO_2_/ZnO (GSZ3); (**c**) Response curves of GSZ1, GSZ2, GSZ3, and GSZ4 (12 h psHT, 24 h psHT, 48 h psHT, 72 h psHT, respectively and then calcination) for different H_2_S gas concentrations; (**d**) Normalized responses of GQDs/SnO_2_/ZnO with H_2_S gas concentrations; (**e**) Gas responses of GSZ3 to different VOCs at 25 °C; (**f**) Selectivity of GSZ3 based on PCA method. (**g**) Responses of pure SnO_2_, Co,N-GQDs, and Co,N-GQDs/SnO_2_ to 100 ppm H_2_S at various temperatures; (**h**) Dynamic H_2_S response transition of pure SnO_2_ (312 °C), N-GQDs/SnO_2_ (260 °C) and Co,N-GQDs/SnO_2_ (260 °C) with the concentration range of 2–100 ppm; (**i**) Selectivity of pure SnO_2_, N-GQDs/SnO_2_, and Co,N-GQDs/SnO_2_; (**j**) Sensing performance of Co,N-GQDs/SnO_2_ under repeated exposure to 40 ppm H_2_S; (**k**) 100 ppm H_2_S response/recovery time of Co,N-GQDs/SnO_2_ at 260 °C; (**l**) Stability for 50 ppb and 40 ppm H_2_S. Reproduced with permission from [83]. Copyright 2020, American Chemical Society.

GQDs-functionalized porous and hierarchical SnO_2_ quantum nanoparticle/ZnO nanostructure (GQDs/SnO_2_/ZnO) were directly self-assembled on digital integrated electrodes through post-synthetic humidity treatment (psHT), with high controllability and repeatability (Figure 19a,b) [83]. Compared with the original ZnO and SnO_2_/ZnO sensors, GQDs/SnO_2_/ZnO nanostructure had high selectivity, high response (15.9 for 0.1 ppm H_2_S), and fast response/recovery time at room temperature (14/13 s) for H_2_S and other interfering gases (Figure 19c–e). The strong synergistic effect between p-type GQDs and n-type SnO_2_ and ZnO, as well as n-p-n heterojunction, effectively amplified the resistance changes caused by oxygen adsorption changes. In the absence of H_2_S gas, a sufficiently high resistance was observed, while the resistance was extremely low when exposed to H_2_S gas. In addition, the sensing characteristics of GQDs/SnO_2_/ZnO sensor was analyzed by principal component analysis (PCA), and it was found that the comprehensive effect of GQDs/SnO_2_/ZnO heterointerfaces helped to improve the selectivity of sensors (Figure 19f). Therefore, sensors with uniformly distributed GQDs and SnO_2_ quantum nanoparticles on 2D nanosheets had great potential for identifying trace target gas in complex environments, especially for noninvasive exhaled diagnosis. In our group, a H_2_S sensor based on Co,N-GQDs/SnO_2_ was prepared by solvothermal method, in which Co,N-GQDs with carboxyl and hydroxyl groups on the surface could coordinate with SnO_2_ nanospheres [119]. At 260 °C, the sensor’s optimal response to 100 ppm H_2_S was about 37.3, which was more than twice the response of pure SnO_2_ at 312 °C (Figure 19g,h). In addition, Co,N-GQDs/SnO_2_ exhibited excellent selectivity, good reproducibility, fast response/recovery time (5/11 s to 100 ppm), and H_2_S detection capability at ppb levels (1.18 to 50 ppb H_2_S) (Figure 19i–l). Co,N-GQDs/SnO_2_ exhibited excellent sensing characteristics, which were mainly attributed to the increase in surface active sites and the electrical modulation of Co,N-GQDs (Equations (13) and (14)).

2H_2_S _(gas)_ + 3O_2_^−^_(ads)_ → 2SO_2(gas)_ + 2H_2_O_(gas)_ + 3e^−^ (13)

H_2_S _(gas)_ + 3O^−^_(ads)_ → SO_2(gas)_ + H_2_O_(gas)_ + 3e^−^ (14)

### 4.5. Isopropanol

Isopropanol is a widely used solvent and an intermediate in pharmaceutical, pesticide, and electronic industries. When exposed to concentrations exceeding 400 ppm, it is extremely harmful to the human body, and can cause dizziness, vomiting, swelling, and internal bleeding [157]. Therefore, the detection of isopropanol is crucial, and this section focuses on the available GQDs-based isopropanol sensors.

**Figure 20:**
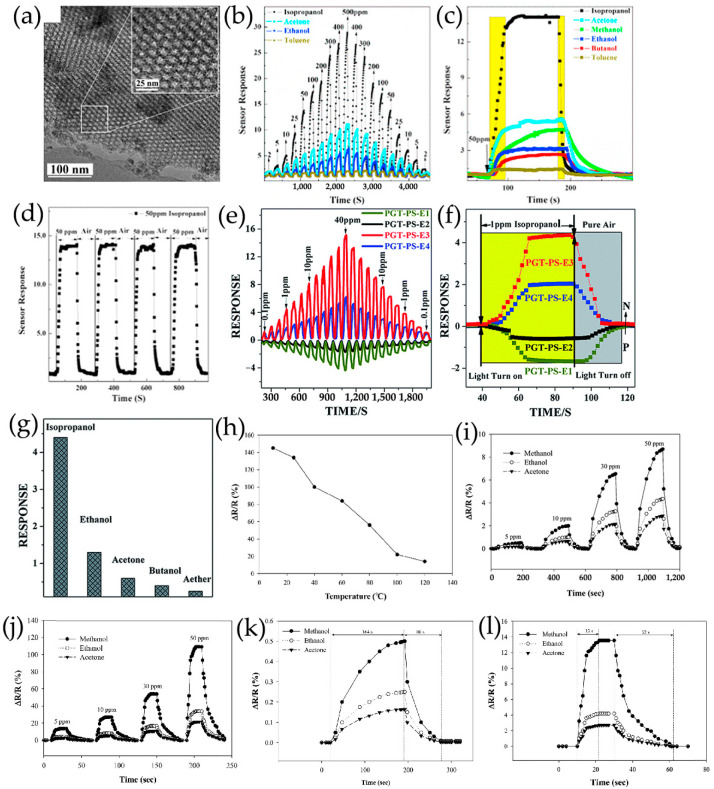
(**a**) TEM image of GQDs/TiO_2_ film; (**b**) Response curves of GQDs/TiO_2_ at various gas concentrations; (**c**) Response curves of GQDs/TiO_2_ sensor to 50 ppm VOCs; (**d**) Response and recovery curves to 50 ppm isopropanol gas. (**e**) Response curves of different PtO_x_/GQDs/TiO_2_ film sensors at different concentrations of isopropanol gas; (**f**) Response/recovery curves of different PtO_x_/GQDs/TiO_2_ to 1 ppm isopropanol gas; (**g**) Selectivity of PGT-PS-E3 to 1 ppm VOCs with oxygen functional group. (**h**) Sensitivity of N-GQDs/PEDOT-PSS to 50 ppm methanol varies with temperature; Dynamic responses (**i**) of original PEDOT-PSS, and (**j**) N-GQDs/PEDOT-PSS to different concentrations of methanol, ethanol, and acetone at room temperature; Response/recovery times of (**k**) original PEDOT-PSS and (**l**) N-GQDs/PEDOT-PSS to 100 ppm of methanol, ethanol, and acetone at room temperature. Reproduced with permission from [102,112,123]. Copyright 2016, Elsevier; copyright 2017, 2015, Royal Society of Chemistry.

Based on crystalline nanoporous GQDs/TiO_2_ thin films, an efficient and convenient sensing system was established by in situ synthesis on the sensing device with water vapor hydrothermal treatment at 120 °C for VOCs detection at room temperature [123]. Water-soluble GQDs were successfully loaded into the thin film with the best density, forming a new type of GQDs/TiO_2_ heterojunctions. Compared with the pristine TiO_2_ thin film, GQDs/TiO_2_ obtained a more ordered nanostructure, smaller crystal size, and larger surface area, making it an ideal candidate for preparing highly sensitive gas sensors. Especially for isopropanol vapor, GQDs/TiO_2_ sensing films showed highly sensitive, fast, and reversible response. The best sensing performance was obtained at 1.6 wt% GQDs, with a response value of 13.8 for isopropanol at 50 ppm, and the response/recovery time of approximately 18/11 s at room temperature (Figure 20a–d). A novel TiO_2_ thin film loaded with PtO_x_ and GQDs was synthesized in situ on a sensor device by water vapor hydrothermal and oxygen plasma methods. The film had adjustable VOCs sensing behavior under visible light irradiation at room temperature [102]. Through oxygen–plasma treatment, the size and oxidation state of GQDs were modified to control the interaction between TiO_2_, and GQDs still retained the sp^2^ hybridized graphene network, exhibiting excellent conductivity and structure stability of graphene. Under visible light irradiation, PtO_x_/GQDs/TiO_2_ were exposed to a V °C concentration range of 0.1–40 ppm and their sensing properties were investigated. Interestingly, as a function of oxygen–plasma treatment, they demonstrated a reversible change of sensing behavior from p-type to n-type of oxygen-functionalized VOCs, as well as p-type sensing properties of aromatic VOCs in the whole process (Figure 20e–g). In addition, PtO_x_/GQDs/TiO_2_ thin film exhibited high sensitivity to isopropanol gas at room temperature, with a response value of up to 4.4 to 1 ppm and a response time as short as 9 s. The functional modification of PtO_x_/GQDs/TiO_2_ thin film played a crucial role in the interaction with VOCs and led to changes in bandgaps.

### 4.6. Methanol

As an important chemical raw material, methanol is widely used in pigments, medicine, organic synthesis, and clean liquid fuel. However, methanol is toxic and harmful to human health, particularly when present in blood and the nervous system. Therefore, it is of great significance to study and develop a methanol sensor that is sensitive, reliable, cost-effective, portable, and selective [158]. Based on N-GQDs/PEDOT-PSS, an efficient and simple sensing system was prepared by dripping on the interdigital Au electrodes on a large silicon substrate with high uniformity, which was characterized for VOCs sensing at room temperature [112]. N-GQDs/PEDOT-PSS sensing system exhibited a highly sensitive, selective, fast, and reversible response to 1–1000 ppm methanol at room temperature. At a low concentration of 50 ppm methanol, its methanol sensing performance was 13 times that of the original PEDOT-PSS. It also had a fast response/recovery time (12/32 s), excellent selectivity and room temperature stability (Figure 20h–l). The methanol-sensing mechanisms of N-GQDs/PEDOT-PSS could be attributed to direct charge transfer and a swelling process.

### 4.7. Trimethylamine

**Figure 21:**
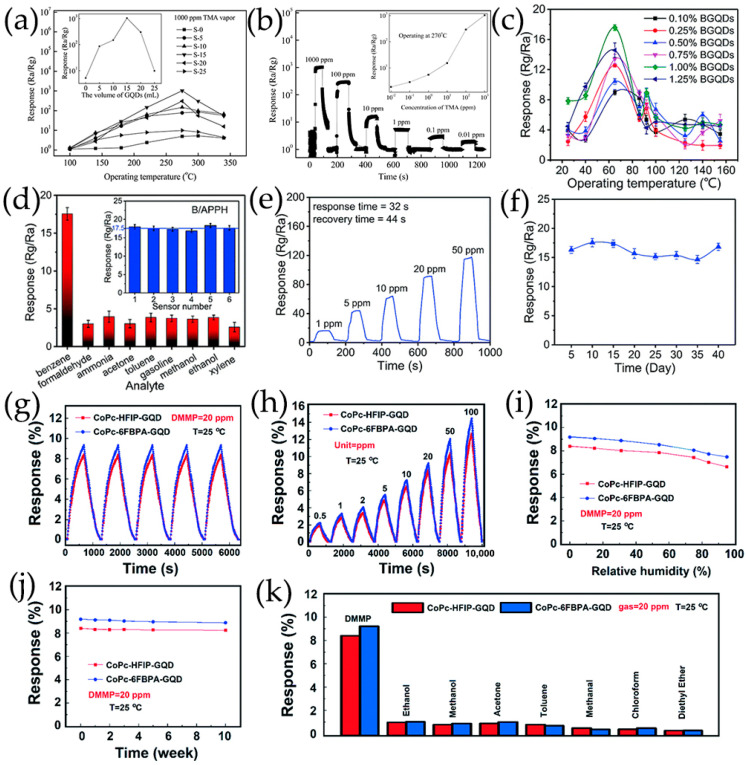
(**a**) Response of GQDs/α-Fe_2_O_3_ to 1000 ppm TMA vapor at various temperature; (**b**) Response transients of GQDs/α-Fe_2_O_3_ (S-15) sensors to TMA vapor at 270 °C. (**c**) Relationship of response-operating temperature to 1 ppm benzene based on BI-AL and B-GQDs; (**d**) Response of B/APPH to different gases at 65 °C; (**e**) Response and recovery time of benzene at 65 °C; (**f**) Benzene stability of B/APPH. Cyclic (**g**), concentration (**h**), humidity (**i**), and time impact curves (**j**) of CoPc-HFIP/GQDs and CoPc-6FBPA/GQDs to 20 ppm DMMP gas at room temperature; (**k**) Selectivity of CoPc-HFIP/GQDs and CoPc-6FBPA/GQDs for 20 ppm DMMP gas at room temperature. Reproduced with permission from [84,101,108]. Copyright 2016, Elsevier; copyright 2018, 2021, Royal Society of Chemistry.

Trimethylamine (TMA) is a hazardous gas with a pungent, fishy, ammoniacal odor, which can cause a series of health problems such as headache, eye irritation, breathing difficulty, pulmonary edema, nausea, and irritation of the upper respiratory system once a certain amount of TMA is inhaled [159]. The maximum allowable exposure duration to TMA is as low as 15 min with a concentration of <15 ppm [160]. In addition, monitoring TMA concentration is an effective method for measuring the freshness of seafood, as it can be released from decayed meat and seafood products. Hence, it has crucial practical significance to develop a convenient and reliable real-time detection method for low concentrations of TMA to ensure the safety of human health, and this section focuses on TMA sensors using GQDs-based materials reported at present. GQDs/α-Fe_2_O_3_ composites were prepared by a simple hydrothermal method in one step [84]. The influence of GQDs content on the morphology and gas sensitive responses of the composites was studied, indicating that GQDs/α-Fe_2_O_3_ composite sensor had high response and selectivity to TMA. GQDs/α-Fe_2_O_3_ response to 1000 ppm and 0.01 ppm TMA at 270 °C reached 1033.0 and 1.9, respectively, which was 187.8 times that of pure α-Fe_2_O_3_ to 1000 ppm TMA. The response/recovery times for 0.01 ppm TMA were only 6/4 s, and those of 1000 ppm TMA vapor were 11/24 s, indicating that TMA vapor could easily react with oxygen on the surface of composites (Equation (15)) (Figure 21a,b). Small-sized GQDs/α-Fe_2_O_3_ nanoparticles could effectively maintain their large specific surface area and provide more active absorption sites, which were conducive to improving the gas-sensitive performance of composites.

4N(CH_3_)_3 (gas)_ + 21O_2_^−^_(ads)_ → 2N_2(gas)_ + 18H_2_O_(gas)_ + 12CO_2(gas)_ + 21e^−^ (15)

### 4.8. Acetic Acid

When handling silicone sealers, acetic acid vapor may corrode workers’ teeth [161]. The concentrations of acetic acid increased from 48 ppb (healthy) to 85 and 170 ppb in the breath of people suffering from cystic fibrosis and gastroesophageal reflux, making acetic acid as a potential breath marker [162]. Acetic acid is also one of the VOCs in packaged beef that has been linked to the amination of Salmonella [163], and during processing of coffee beans and chocolate, it is a key tracer for aroma development and product quality [164]. Therefore, real-time monitoring of acetic acid is very important, and this discussion covers acetic acid sensors reported using GQDs-based composites. GQDs/ZnO composites with different GQDs contents were synthesized by hydrothermal method [110]. The effects of GQDs on the gas sensitivity and selectivity of ZnO-based sensors were studied. It was found that the optimal composition of GQDs/ZnO composites could work at room temperature, and have better response and selectivity for acetic acid gas than pure ZnO sensors. However, the response of the sensor decreased if the content of GQDs in composite was too high. GQDs/ZnO composite sensors could detect 1 ppm of acetic acid vapor at room temperature. The response/recovery times for 1 ppm acetic acid were 11 s and 12 s, respectively. At room temperature, the GQDs/ZnO composite sensors had a response of 1642 to 1000 ppm HCHO, demonstrating excellent selectivity to acetic acid (Equation (16)).

CH_3_COOH_(ads)_ + 2O_2_^−^_(ads)_ → 2CO_2(gas)_ + 2H_2_O_(gas)_ + 2e^−^ (16)

### 4.9. Benzene

Benzene, as one of the main VOCs in residential space and workplaces, is widely concerning because of its toxicity and carcinogenicity, which usually originates from petrochemical processes, household items, construction materials, vehicle exhaust fumes, and smoking. It is reported that even being exposed to a very low concentration of benzene (1–2 ppm or lower) could increase the risk of leukemia and cancer, especially for vulnerable population groups [165]. Moreover, benzene as a biomarker in exhalation is associated with lung cancer, so detecting low concentration benzene might be a good strategy to early diagnose lung cancer through breath analysis as a non-invasive diagnostic strategy [166]. Therefore, real-time detection of low concentration benzene is of great significance. The available benzene sensors based on B-GQDs/Ag-LaFeO_3_ p-p heterojunctions were synthesized using microwave chemical method [108]. The bandgap of B-GQDs and Ag-LaFeO_3_ was well matched, which promoted the separation of electron–hole pairs and enhanced the carrier transport ability. Ag, as a good catalyst, could promote the oxidation of benzene to CO_2_ and H_2_O in gas sensitive reactions. In addition, Ag-LaFeO_3_ was modified by benzene molecular imprinting to optimize selectivity of B-GQDs/Ag-LaFeO_3_ (B/APPH), which could specifically recognize benzene during detection. The molar ratios of benzene template to Ag-LaFeO_3_ were 3:10, 4:10, 5:10, and 6:10. The gas sensing performance of Ag-LaFeO_3_ and B/APPH was compared. Ag-LaFeO_3_ had a response of 18.5 for benzene and less than 5 for other interfering molecules at the optimal operating temperature of 125 °C. After adding 1.00% B-GQDs, the optimal operating temperature was reduced to 65 °C, and B/APPH had high response (17.5), good selectivity, repeatability, and accuracy, which could detect benzene as low as 1 ppm (Figure 21c–f). The heterojunction between B-GQDs and Ag-LaFeO_3_ improved carrier transport and lowers working temperature, while maintaining high sensing response and selectivity.

### 4.10. DMMP

Sarin gas is one of the deadliest and most famous chemical warfare agents (CWAs). In the field of public safety and military affairs, it is very important to detect CAWs such as sarin in time [167]. However, because of its strong toxicity, it cannot be directly used as a chemical detection object for the experimental test of gas sensors. Dimethyl methylphosphonate (DMMP) has the advantages of nontoxicity, similar molecular structure and functional groups, so it is the ideal gas to simulate sarin in experiments [168]. In this section, a DMMP gas sensor based on cobalt phthalocyanine (CoPc) derivative/GQDs hybrid materials was developed [101]. CoPc derivatives containing hexafluoroisopropanol (HFIP) and hexafluorbisphenol A (6FBPA) were prepared, and then combined with GQDs by π-π bonds to from hybrid materials. CoPc/GQDs exhibited good gas response properties at room temperature. Within the same response time (600 s), CoPc-6FBPA/GQDs had better response performance than CoPc-HFIP-GQDs. The response of CoPc-6FBPA/GQDs and CoPc-HFIP-GQDs for 20 ppm DMMP was 9.3 and 8.4, respectively, and the recovery time of CoPc-6FBPA/GQDs and CoPc-HFIP-GQDs for 20 ppm DMMP was 620 s and 640 s, respectively (Figure 21g–k). This hybrid material had good reproducibility, stability, and selectivity, with a minimum response concentration of up to 500 ppb for DMMP. There were two main reasons for this performance: one was the formation of strong hydrogen bonds between HFIP/6FBPA and DMMP, and the other was that the incorporation of GQDs greatly improved the conductivity of hybrid materials through π-π bonds with CoPc derivatives. In addition, the problem of slow recovery of CoPc derivatives was solved by means of laser-assisted irradiation. Therefore, these CoPc derivatives/GQDs hybrid materials were expected to be feedstocks for sarin gas sensor.

### 4.11. NO_2_

Nitrogen dioxide (NO_2_) is a poisonous gas with a pungent smell that came from vehicle exhausts, fossil fuel burning, and industrial production activities [169]. The emission of NO_2_ will not only cause acid rain and have serious negative effects on water, land, and artificial ecosystems, but also lead to photochemical haze on the ground, which can cause great harm to the environment and human health [58]. Therefore, in order to protect the environment and human health, it is necessary to conduct highly sensitive and selective detection of NO_2_ concentrations. The available NO_2_ sensors developed using GQDs-based composites are shown below.

Li et al. [105] proposed a new method for manufacturing Si-based gas detectors, which used vertically aligned Si nanowire (SiNW) arrays as skeletons and platforms, with large surface areas and sufficient gas molecule diffusion gaps. Chemically inert GQDs were uniformly modified through a vacuum film to protect SiNW from oxidation and promote carriers/analytes interactions (Figure 14j–l). Then, the radial core-shell structures of GQDs/SiNW array were assembled into a resistance-based gas detection system, and NO_2_ was used as a model analyte for evaluation. Compared with bare SiNW arrays, GQDs/SiNW arrays exhibited higher sensitivity (approximately 140 for 500 ppm NO_2_), faster recovery speed, better stability, and repeatability. At room temperature, it could effectively identify trace levels of NO_2_ as low as 10 ppm, and the sensitivity of GQDs/SiNW array did not decrease even after two weeks of storage in the air. Therefore, the GQDs/SiNW array could serve as a framework for further specific modification to expand the sensing range.

**Figure 22:**
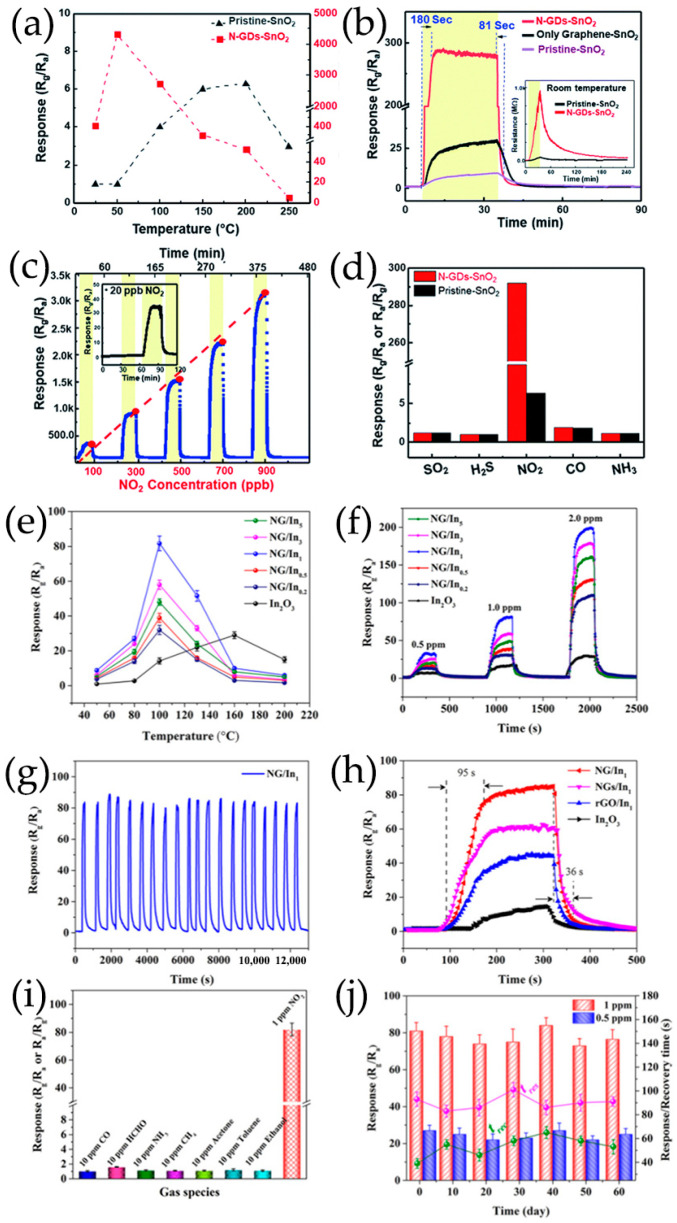
(**a**) Sensing response of SnO_2_ and N-GQDs/SnO_2_ to 100 ppb NO_2_ gas at various temperatures; (**b**) Comparison of response/recovery time at 150 °C; (**c**) NO_2_ sensing response of N-GQDs/SnO_2_ at 150 °C; (**d**) Selectivity of SnO_2_ and N-GQDs/SnO_2_. (**e**) Response–temperature curves of N-GQDs/In_2_O_3_ (NG/In_x_, x represents N-GQDs concents) and 3DOM In_2_O_3_; (**f**) Response–recovery curves of NG/In_x_ and 3DOM In_2_O_3_; (**g**) 1 ppm NO_2_ repeatability of NG/In_1_; (**h**) 1 ppm NO_2_ response curves of NG/In_1_, rGO/In_1_, NS/In_1_, and 3DOM In_2_O_3_; (**i**) Selectivity of NG/In_1_; (**j**) Stability of NG/In_1_ to NO_2_. Reproduced with permission from [70,118]. Copyright 2020, Royal Society of Chemistry; copyright 2020, American Chemical Society.

0D N-GQDs/SnO_2_ quantum dot heterostructures were prepared using a facile wet chemical method. Within the entire temperature range of 25 °C to 250 °C, the heterostructure had a higher response to 100 ppb NO_2_, which was superior to the original SnO_2_, pure GQDs/SnO_2_, and graphene-based gas-sensors [118]. After surface modification with N-GQDs, N-GQDs/SnO_2_ showed enhanced response (*R_g_*/*R_a_* = 292) and shorter response/recovery time (180/81 s) to 100 ppb NO_2_ gas at 150 °C. As the temperature was reduced to 50 °C, the response value increased to 4336, while the temperature increased to 200 °C, the response of original SnO_2_ only increased to 6 (Figure 22a–c). In addition, N-GQDs/SnO_2_ could detect NO_2_ at an ultralow concentration of 20 ppb, had a high response, and showed selectivity to NO_2_ than other gases with reliable long-term stability (Figure 22d). The improved gas sensitivity of NO_2_ was due to synergistic effect at the interface, which enhanced electron transfer from SnO_2_ to N-GQDs, as well as enhanced adsorption of NO_2_ on N-GQDs surface, especially at low temperatures. Moreover, due to the large surface area, more active sites, and better nanoscale interfaces, the 0D morphology also helped to improve sensing performance. Therefore, the unique N-GQDs/SnO_2_ heterogeneous nanostructure was expected to be used for ultrasensitive sensor applications.

N-GQDs/3DOM In_2_O_3_ with different N-GQDs loading contents were constructed successfully, and their NO_2_ response performance were studied [70]. Compared with pure 3DOM In_2_O_3_, rGO/3DOM In_2_O_3_, and N-doped graphene sheets (NS)/3DOM In_2_O_3_, N-GQDs/3DOM In_2_O_3_ exhibited higher NO_2_ sensing performance, characterized by fast response and recovery, high response, low detection limit, good selectivity and repeatability, long-term stability, and low operating temperature. When NO_2_ concentration was 1 ppm, N-GQDs/3DOM In_2_O_3_ showed a maximum response value of 81.7, which was approximately 5.8 times that of the original 3DOM In_2_O_3_ at 100 °C (Equations (17) and (18)).

Compared with 3DOM In_2_O_3_, the optimal operating temperature of N-GQDs/3DOM In_2_O_3_ was reduced from 160 to 100 °C. At optimal operating temperature, the response/recovery times of N-GQDs/3DOM In_2_O_3_ were about 95 and 36 s, respectively. In addition, the optimized N-GQDs/3DOM In_2_O_3_ sensor for NO_2_ response had low detection limit of 100 ppb, excellent selectivity, and good long-term stability without significant fluctuations in response within 60 days (Figure 22e–j). The formation of heterojunctions between N-GQDs and 3DOM In_2_O_3_, the unique 3DOM structures and the doping N atoms in N-GQDs were important factors to improve NO_2_ response.

NO_2(gas)_ + e^−^ → NO_2_^−^_(ads)_ (17)

NO_2(gas)_ + O^−^_(ads)_ → NO_3_^−^_(ads)_ (18)

N-GQDs/ZnO were successfully synthesized using the hydrothermal method [114]. The gas sensing test results showed that N-GQDs/ZnO doped with 2 mL N-GQDs exhibited excellent sensing properties for NO_2_. The sensor based on the optimal N-GQDs/ZnO significantly reduced the working temperature from 160 °C to 100 °C, and the response to 5 ppm NO_2_ significantly improved by approximately 11.6 times. The detection limit was as low as 0.1 ppm. In addition, this method had good reproducibility, selectivity, and stability for NO_2_ detection. The improvement in gas sensitivity was mainly due to the synergistic effect of N-GQDs/ZnO heterojunction, highly active N atoms, and oxygen vacancies (Equations (17) and (18)). The interfacial heterojunction between N-GQDs and ZnO expanded resistance modulation, and the doping of highly active N atoms enhanced the electron transfer from ZnO to N-GQDs and the adsorption of NO_2_ molecules.

**Figure 23:**
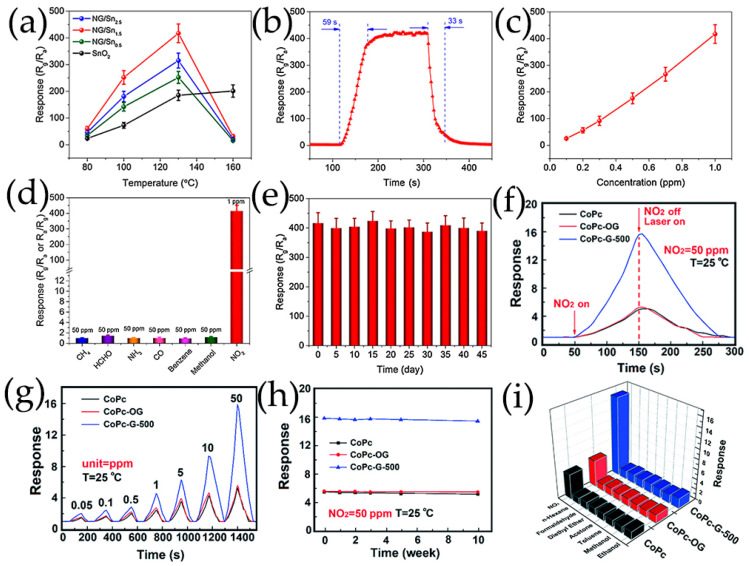
(**a**) The 1 ppm NO_2_ response of SnO_2_ and N-GQDs/SnO_2_ (NG/Sn_x_, x represents N-GQDs theoretical ratio) at various temperatures; (**b**) NG/Sn_1.5_ response curve to 1 ppm NO_2_ at 130 °C; (**c**) NG/Sn_1.5_ response values at different NO_2_ concentrations; (**d**) NG/Sn_1.5_ selectivity for NO_2_; (**e**) NG/Sn_1.5_ response to 1 ppm NO_2_. (**f**) Response curves, (**g**) Concentration–effect curves, (**h**) Time impact curves of CoPc, CoPc-OG (CoPc/O-GQDs) and CoPc-G-500 (CoPc-COOH with GQDs at 500 °C for 6 h with a mass ratio of 4:1) at room temperature; (**i**) Selectivity of CoPc, CoPc-OG, and CoPc-G-500 for 50 ppm NO_2_ and other 100 ppm analytes. Reproduced with permission from [58,100]. Copyright 2021, Elsevier; copyright 2021, Royal Society of Chemistry.

Using a method of sulfuration-oxidation-impregnation-calcination, a new SnO_2_ hierarchical hollow cube consisting of ultrathin mesoporous nanosheets uniformly modified by N-GQDs were successfully prepared [100]. The sulfuration-oxidation method could not only produce homogeneous hierarchical hollow cubic structure made of ultrathin SnO_2_ nanosheets, but also generate large quantities of mesopores on nanosheets. Its stable gas adsorption and diffusion channels, large specific surface area, and rich nanoscale grain boundaries were conducive to improving the sensing performance of NO_2_. In addition, the construction and N doping of 0D/3D N-GQDs/SnO_2_ could greatly increase the spatial charge modulation depth and NO_2_ adsorption active sites of composites, thus improving the NO_2_ sensing properties (Equations (17) and (18)). The optimized N-GQDs/SnO_2_ sensor had excellent detection capabilities for low concentration NO_2_ (100 ppb) (*R_g_*/*R_a_* = 25.3) and showed improved response to 1 ppm NO_2_ (*R_g_*/*R_a_* = 417), which was about 2.2 times that of pure SnO_2_ at 130 °C. In addition, the optimized N-GQDs/SnO_2_ sensor had rapid response/recovery speed (59/33 s), good selectivity, repeatability, and long-term stability for NO_2_ gas at low operating temperature (130 °C) (Figure 23a–e).

Utilizing the excellent detection performance of metal phthalocyanine (MPc) and GQDs for NO_2_, anchored by GQDs on the surface of MPc nanofibers via π-π stacking, a charge transfer conjugate was formed, and a gas sensor based on MPc/GQDs was obtained [58]. Under a certain proportion of components, the response to NO_2_ was significantly enhanced, which was much better than their individual response to NO_2_ at room temperature. The incorporation of GQDs greatly improved the conductivity of phthalocyanine fibers, resulting in a faster CoPc/GQDs response. The response value of CoPc/GQDs to 50 ppm NO_2_ within 100 s was 15.8 times that of CoPc (Figure 23f,g). In addition, the reproducibility, selectivity, and stability of CoPc/GQDs were greatly improved, with the lowest response concentration as low as 50 ppb (Figure 23h,i). The problem of slow recovery of MPc was solved by using ultra-low power laser irradiation.

A novel magnetic graft reduction of 2-naphthalenesulfonic to GOQDs (Fe_3_O_4_-rGOQD-naphthalene-2-SO_3_H) was prepared by a five-step method. Fe_3_O_4_ (X = 99, 95, 90, 85 wt% ) with different weight percentages was used as NO_2_ gas sensors, which had high sensitivity, reversibility, and selectivity [104]. The concentration range of 2.5–50 ppm NO_2_ was chosen for exploring the efficiency of Fe_3_O_4_-rGOQD-naphthalene-2-SO_3_H as a NO_2_ sensor at 20% humidity and room temperature. Fe_3_O_4_-rGOQD-naphthalene-2-SO_3_H with 15% loading of rGOQD-naphthalene-2-SO_3_H showed the best response while the concentration of NO_2_ was 10 ppm. When NO_2_ concentration increased from 2.5 to 50 ppm, the sensitivity of the optimal Fe_3_O_4_-rGOQD-naphthalene-2-SO_3_H increased from 65 to 130. The Fe_3_O_4_-rGOQD-naphthalene-2-SO_3_H sensor could spontaneously recover to their initial states by flowing N_2_ gas without thermal assistance or chemical reaction, and the response/recovery time was 25/97 s. The results showed that the Fe_3_O_4_-rGOQD-naphthalene-2-SO_3_H exhibited improved responsiveness, detection range, and optimum temperature compared to other works. The relatively high surface area and unique electronic properties of Fe_3_O_4_-rGOQD-naphthalene-2-SO_3_H improved its application as a gas sensor compared to pure Fe_3_O_4_, demonstrating that chemical modification of rGO with sulfonic acid and creation of quantum dots on the surface of rGO is an effective way to improve the performance of rGO for gas-sensing applications.

An alternating stacking 3D structure based on MoS_2_/rGO/GQDs ternary hybrids was prepared by anchoring MoS_2_ and GQDs on rGO nanosheets, improving the gas-sensitive properties of NO_2_ at room temperature [122]. The introduction of GQDs can prevent the aggregation of nanosheets in the mixing process of rGO and MoS_2_, significantly improve the uniform distribution of rGO and MoS_2_ nanosheets, and provide abundant reaction sites for NO_2_ gas adsorption, which was conducive to electron transport and further improve the gas-sensitive properties of MoS_2_/rGO/GQDs hybrids. rGO nanosheets were used as the carrier transport channels and substrates for the growth of MoS_2_ nanoflowers. The prepared sensor based on MoS_2_/rGO/GQDs showed a response of 23.2% to 50 ppm NO_2_ at room temperature and a response of 15.2% at as low as 5 ppm NO_2_. The response and recovery time was stable at 150 s. In addition, the prepared MoS_2_/rGO/GQDs had excellent repeatability and gas selectivity. When exposed to some typical interfering gases, the sensitivity of MoS_2_/rGO/GQDs to NO_2_ was more than 10 times that of other gases. The synergistic effects of 3D nanostructure, heterojunction, and GQDs in MoS_2_/rGO/GQDs enabled it to have excellent gas sensing capabilities (Equation (19)).

2NO_2(gas)_ + O_2_^−^_(ads)_ + e^−^ → 2NO_3_^−^_(ads)_ (19)

### 4.12. NO

**Figure 24:**
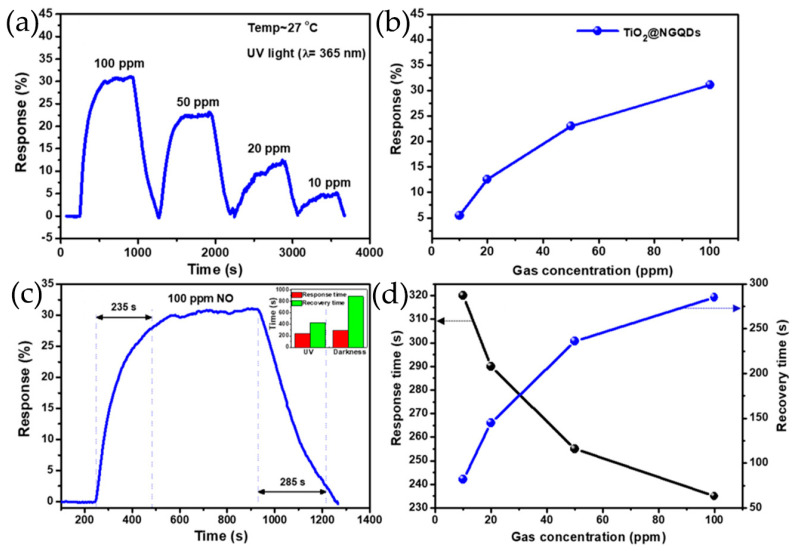
(**a**) Sensing performance of TiO_2_/NGQDs measured under UV irradiation at room temperature; (**b**) Response as a function of NO gas concentration; (**c**) Response/recovery time measured from the response curve of 100 ppm NO gas; (**d**) Changes of response/recovery time values with NO gas concentration. The illustration in (**c**) is a comparison of response and recovery time of TiO_2_/N-GQDs to 100 ppm NO gas under dark and UV irradiation. Reproduced with permission from [113]. Copyright 2020, American Chemical Society.

Nitric oxide (NO) is a type of exhaust gas mainly produced by the burning of fossil fuels in automobiles and factories, which has adverse effects on the environment and human health [170]. It plays an important role in the formation of photochemical smog in air, airpocalypse, and acid rain [171]. It is also central to the formation of fine particles (PM) and ground-level ozone, which adversely impacts human health and causes damage to buildings [172]. Moreover, monitoring the concentration of inhaled and exhaled NO gas was crucial for identifying cardiac valve surgery and airway inflammation, respectively. Thus, it was very commendable to develop a high-sensitivity NO gas sensor. A high-performance room-temperature NO gas sensor activated by UV light based on N-GQDs modified TiO_2_ nanoplate hybrid structure was demonstrated [113]. TiO_2_ used in the form of {001} crystal planes exhibited the shape of rectangular nanoplate, exhibiting high reactivity in adsorbing reactive oxygen species. The precursor was graphitized by hydrothermal treatment and N-GQDs layers were grown on TiO_2_ nanoplates. The introduction of N-GQDs on TiO_2_ surface significantly improved the efficiency of gas and carrier exchange, charge carrier separation and transport, and oxygen vacancy, and finally improved the sensing performance (Equations (20) and (21)). At room temperature, the TiO_2_/N-GQDs hybrid sensor had a 12.0% response to 100 ppm NO, which was 4.8 times that of the original TiO_2_ nanoplates. In addition, the sensing performance of TiO_2_/N-GQDs sensor driven by UV light was 2.6 times higher than that without UV irradiation. Under UV (λ = 365 nm) irradiation at room temperature, the response of the hybrid structure increased to 31.1% when NO was 100 ppm (Figure 24a,b). On the other hand, at the optimal operating temperature of 250 °C, the customized operating temperature produced a response of 223%. The response and recovery time of TiO_2_/N-GQDs in the dark were 298 and 890 s and under UV irradiation were 235 and 285 s, respectively (Figure 24c,d). Under UV irradiation, the recovery time of TiO_2_/N-GQDs was shortened by 605 s. The enhancement of performance under UV irradiation was due to the formation of TiO_2_/N-GQDs-based heterojunctions, which effectively generated and separated photogenerated charge carriers, and prevented the recombination of electron–hole pairs.

NO_(gas)_ + e^−^ → NO^−^_(ads)_ (20)

NO^−^_(ads)_ + O_2_^−^_(ads)_ + 2e^−^ → NO_(gas)_ + O^−^_(ads)_ (21)

## 5. Conclusions and Future Perspectives

Obviously, the development of chemiresistive gas sensors based on GQDs and their composites has become one of the research directions in various applications, including environmental monitoring, healthcare and safety. This paper reviews the research progress in gas sensors based on GQDs in recent years, including synthesis strategies of GQDs and their composites, the influence of surface and intrinsic sensing properties of GQDs, heterojunctions, morphologies, doping/de-doping and swelling process of polymers on the improvement of gas sensing properties, as well as the sensing characteristics arising therefrom. The gas sensing key points of chemiresistive gas sensors based on GQDs are summarized as follows:

(1) The excellent electronic mobility, high specific surface area, minute size, surface functionalization, and doping with heteroatoms of GQDs enables better driving force for gas diffusion, more oxygen active sites for the interaction, and easily introduced into other functional materials to form high-quality composite materials, which can remarkably enhance the sensing performance.
(2) According to the reported literature on GQDs-based chemiresistive gas sensors, the addition of GQDs can effectively decrease the operating temperature of the sensors. 87.5% of the sensors can achieve the excellent sensing performance below 150 °C, and almost 80% can work below 100 °C or at room temperature, showing enormous application potential.
(3) The formation of heterojunctions between GQDs and metals, metal oxides, MPc, polymers, metal sulfides, and SiNW results in the redistribution of carriers at the interface, reducing the enthalpy and activation energy needed for adsorbing target gas molecules, thereby improving transduction between internal grains.
(4) The morphology of sensing materials also plays an important role in the improvement of gas sensing properties. It can expand the specific surface area of the materials, provide more active reaction sites, favorable adsorption, and diffusion channels, so that the analytes can be quickly adsorbed and desorbed.

Although remarkable progress has been made in developing novel chemiresistive gas sensors based on GQDs, the number of chemiresistive gas sensors using GQDs and its composites remains limited. There are still many challenges and issues in achieving high response, quick response/recovery speed, excellent selectivity, and long-term stability:

(1) One of the key challenges is the exploration of synthetic strategies of novel GQDs with specific properties. The shape, size, surface characteristic, and inner structure have significant impacts on the gas sensing performance. Currently, the relatively single precursors used for the synthesis of GQDs as chemiresistive gas-sensing materials and the low purity of the desired GQDs limit the improvement of gas-sensing performance, thereby limiting their applicability in real-life applications.
(2) The gas-sensitive mechanisms of GQDs-based nanocomposites with different sizes and morphologies are introduced to explain their sensing characteristics. However, there is no clear explanation as to why the same GQDs based composites with similar precursors, size, morphologies, and heterojunctions exhibit significantly different sensing characteristics. Therefore, the development of in situ characterization technologies and theories of sensing mechanisms are necessary.

To sum up, it is still a great challenge to rationally design advanced GQDs-based sensing materials with excellent sensing characteristics because of the limitations on clearer sensing mechanisms and improved synthesis methods. It is believed that this review will provide a forward-looking direction for researchers and stimulate new ideas to elevate the research of chemiresistive gas sensors to a new level.

## Figures and Tables

**Table 1:** Summary of methods for synthesizing GQDs as chemiresistive gas sensing materials.

Approach	Synthesis Method	Material	Precursors	Reaction Procedure	Refs.
Top-down	Hydrothermal synthesis	GQDs	Graphite nanopowder	(1) graphite oxide: modified Hummers method; (2) hydrothermal treatment of graphite oxide + hydrazine hydrate at 180 °C for 12 h.	[98]
		N-GQDs	Graphite flake	(1) GO: modified Hummers method; (2) hydrothermal treatment of GO + ammonia at 200 °C for 10 h.	[99]
		N-GQDs	Graphite flake	(1) GO: modified Hummers method; (2) hydrothermal treatment of GO + ammonia at 180 °C for 12 h.	[70,100]
		GQDs	Graphite	(1) GO sheets: modified Hummers method; (2) rGO: GO + hydrazine at 100 °C for 3 h; (3) rGO + H_2_SO_4_ + HNO_3_ under ultrasonication for 12 h; (4) hydrothermal treatment of oxidized rGO sheets with pH 8 at 200 °C for 10 h.	[58,101]
		GQDs	GO quantum dots	(1) hydrothermal treatment of GO quantum dots + hydrazine hydrate at 180 °C for 8 h	[102]
	Solvothermal synthesis	GQDs	Graphite powder	(1) GO: modified Hummers method; (2) solvothermal treatment of GO + DMF at 200 °C for 8 h.	[103]
		rGOQDs	Flake graphite powder	(1) GO: modified Hummers method; (2) GOQDs: solvothermal treatment of GO + DMF at 250 °C for 6 h; (3) rGOQDs: GOQDs + NaBH_4_ at 80 °C for 1 h.	[104]
	Electrochemical exfoliation	GQDs	Graphite powder	(1) GO: modified Hummers method; (2) rGO: GO + ammonia + vacuum filtered, form a free-stranding film; (3) working electrode: rGO film, counter electrode: Pt mesh, reference electrode: saturated calomel electrode, electrolyte: PBS, CV: ±3 V, scan rate: 0.5 V/s.	[105]
		GQDs	Graphite rods	(1) Graphite rods as electrodes, citric acid and KCl as electrolyte, operated at 8 V for 15 min.	[106]
	Liquid-phase exfoliation	GQDs	Multiwalled CNTs (MWCNTs)	(1) ultrasonication of MWCNTs + H_2_SO_4_ + HNO_3_ at 60 °C for 48 h.	[68]
		GQDs	Graphite powder	(1) ultrasonication of graphite powder + H_2_SO_4_ + HNO_3_ at 45 °C for 2 h, then maintained at room temperature for 4 days, heated at 55 °C for 12 h.	[107]
		B-GQDs	Graphite powder	(1) boracic CF: Graphite powder + boric acid; (2) ultrasonication of boracic CF + NMP for 10 h.	[108]
		B, N, Cl-GQDs	Degreasing cotton	(1) carbon fibers (CF): Degreasing cotton in a 1000 °C oven and heated in air for 30 min; (2) B, N, Cl-GQDs: CF + boric acid/HNO_3_/HCl under ultrasonication for 10 h.	[109]
	Physical grinding	S-GQDs	Graphite	(1) graphite + sulfur in an agate jar containing agate balls; (2) Seal the jar and charge it with argon after several charging and discharging cycles, then fix it in a planetary ball mill and stir at 500 rpm for 48 h.	[109]
Bottom-up	Pyrolysis	GQDs	Citric acid	heated at 200 °C for 30 min	[69,84,86]
	Hydrothermal synthesis	GQDs	Citric acid	hydrothermal treatment, 180 °C, 5 h	[110,111]
		N-GQDs	Citric acid + urea	hydrothermal treatment, 160 °C, 4 h	[112,113,114,115]
			Citric acid + urea	hydrothermal treatment, 180 °C, 8 h	[116,117]
			Citric acid + dopamine hydrochloride	hydrothermal treatment, 180 °C, 6 h	[118]
		Co, N-GQDs	Citric acid + urea + CoCl_2_·6H_2_O	hydrothermal treatment, 160 °C, 8 h	[119]
		S, N-GQDs	Citric acid + thiourea	hydrothermal treatment, 160 °C, 4 h	[67,120]
		OH-GQDs	Pyrene	(1) Pyrene + HNO_3_ at 80 °C under stirring for 12 h; (2) hydrothermal treatment, 200 °C, 10 h.	[121]
		GQDs	Benzopyrene	(1) Benzopyrene + HNO_3_ at 80 °C under stirring for 12 h; (2) hydrothermal treatment at 200 °C for 8 h.	[122]

**Table 2:** Summary of synthesis and reaction processes of GQDs-based composites for chemiresistive gas sensing applications.

No.	Materials Category	Sensing Material	Chemical Used and Reaction Processes	Morphology	Target Gas	Ref.
1	Metal oxide/ GQDs composite	SnO_2_/GQDs	(1) Solvothermal treatment of GQDs + DMF + SnCl_4_·5H_2_O + urea at 160 °C for 10 h.	Nano-composites	Acetone	[103]
2		Co, N-GQDs/ SnO_2_	(1) Solvothermal treatment of Co,N-GQDs + SnCl_4_·5H_2_O at 160 °C for 15 h.	Mesoporous microspheres	H_2_S	[119]
3		N-GQDs/SnO_2_	(1) SnO_2_: hydrothermal treatment of PVP + Na_3_C_6_H_5_O_7_·2H_2_O + SnCl_2_·2H_2_O at 180 °C for 12 h; (2) N-GQDs/SnO_2_: ultrasonic impregnation of N-GQDs in SnO_2_	Nanosheets	HCHO	[117]
4		N-GQDs/SnO_2_	(1) SnO_2_: calcination of GO + dibutyltin dilaurate, 500 °C, 2 h; (2) N-GQDs/SnO_2_: ultrasonic impregnation of N-GQDs + SnO_2_	2D mesoporous ultrathin structure	HCHO	[116]
5		N-GQDs/SnO_2_	(1) SnO_2_: heating of tin(IV) bis(acetylacetonate)dichloride + 1,2-hexadecanediol + dibenzyl ether + oleylamine under dry and oxygen-free vacuum conditions; (2) N-GQDs/SnO_2_: ultrasonication and stiring of N-GQDs + SnO_2_	0D heterostructures	NO_2_	[118]
6		N-GQDs/SnO_2_	(1) SnS_2_: hydrothermal treatment of ZnSn(OH)_6_ + ethylene-diaminetetraacetic acid + thioacetamide at 220 °C for 3 h; (2) SnO_2_: calcination of SnS_2_ at 400 °C for 2 h; (3) N-GQDs/SnO_2_: vacuum dried and heating of N-GQDs + SnO_2_ at 250 °C for 2 h	Mesoporous hierarchical hollow cubes	NO_2_	[100]
7		GQDs/ZnO	(1) Hydrothermal treatment of GQDs + Zn(NO_3_)_2_·6H_2_O + NaOH at 180 °C for 12 h	Nano-composites	Acetic acid	[110]
8		N-GQDs/ZnO	(1) Hydrothermal treatment of ethylene glycol + AOT + Zn(CH_3_COO)_2_·2H_2_O + urea + GQDs at 120 °C for 12 h, dried at 60 °C for 12 h and calcined at 400 °C for 2 h	Ultra-thin nanosheets	NO_2_	[114]
9		ZnO/GQDs	(1) ZnO: cylindrical zinc transformed into ZnO under DC electrical current in an air atmosphere; (2) ZnO/GQDs: GQDs dropped onto ZnO	p-n heterojunction	NH_3_	[106]
10		GQDs/ZnO	(1) ZnO: sol–gel method with PMMA latex spheres + Zn(NO_3_)_2_; (2) GQDs/ZnO: GQDs solution spin-coated on ZnO	3DOM	Acetone	[107]
11		GQDs/ZnO	(1) ZnO nanorod thin film: a. ZnO NPs: Zn(CH_3_COO)_2_·2H_2_O + HN(CH_2_CH_2_OH)_2_ + ethanol, heated at 60 °C; then as seed spin-coated on substrates, dried at 200 °C and annealed at 500 °C; b. Pre-seeded substrates + Zn(NO_3_)_2_·6H_2_O + NaOH, annealed at 500 °C; (2) GQDs/ZnO: GQDs dropped on ZnO and dried at 300 °C	Nanorod thin film	Ethanol	[69]
12		N-GQDs/In_2_O_3_	(1) In_2_O_3_: PS template method (citric acid + In(NO_3)3_·4.5H_2_O, vacuum filtration, then dried and heated at 550 °C for 3 h); (2) N-GQDs/In_2_O_3_: hydrothermal treatment of In_2_O_3_ + N-GQDs at 150 °C for 4 h	3DOM	NO_2_	[70]
13		TiO_2_/N-GQDs	(1) TiO_2_: hydrothermal treatment of Ti(OBu)_4_ + HF at 180 °C for 24 h; (2) TiO_2_/N-GQDs: hydrothermal treatment of TiO_2_ + citric acid + urea at 160 °C for 4 h	Nanoplate hybrid structure	NO	[113]
14		GQDs/TiO_2_	(1) GQDs-Ti precursor solution (GQDs + TiCl_4_ + ethanol + F127 polymer + HCl) spin-coated onto sensor device and dried; (2) Sensor device: water vapor hydrothermal treatment, 90% relative humidity, 120 °C, 96 h, then heat treatment at 250 °C for 2 h and 300 °C under N_2_ atmosphere for 1 h	Ordered nanoporous thin film	Isopropanol	[123]
15		N-GQDs/TiO_2_/ graphene foam	(1) Sensor device + graphene foam immersed in Au-Ti precursor solution (TiCl_4_ + N-GQDs + gold solution + HCl) for 30 s, raised for 4 times at the speed of 20 mm/min, then dried at 60 °C for 30 min; (2) Sensor device: water vapor hydrothermal treatment, 95% relative humidity, 150 °C, 72 h, then heat treatment at 573 K for 2 h under N_2_.	3D nanoporous composites	HCHO	[100]
16		GQDs/α-Fe_2_O_3_	(1) Hydrothermal treatment of Fe(NO_3_)_3_·9H_2_O + GQDs + ammonia water at 180 °C for 12 h)	Nano-composites	Trimethylamine	[84]
17		Fe_3_O_4_-rGOQD-naphthalene-2-SO_3_H	(1) FeCl_2_·4H_2_O + FeCl_3_·6H_2_O + rGOQD-naphthalene-2-SO_3_H + ammonia solution at 70 °C for 30 min	/	NO_2_	[104]
18	Metal-metal oxide/GQDs composite	GQDs/Pt-SnO_2_	(1) Spin-coating of GQDs/Pt-Sn precursor solution (SnCl_4_ + PtCl_4_ + PluronicF127 triblock copolymer + GQDs) onto sensor device; (2) Sensor device: water vapor hydrothermal treatment, 120 °C, 96 h, 90% relative humidity, annealing at 300 °C for 2 h	Ordered nanoporous thin film	Acetone	[98]
19	Ternary metal-oxide/GQDs composite	PtO_x_/GQDs/ TiO_2_	(1) Spin-coating of PtOx/GQDs-Ti precursor solution (TiCl_4_ + PluronicF127 triblock copolymer + GQDs + PtCl_4_ + citric acid) onto sensor device; (2) Sensor device: water vapor hydrothermal treatment, 120 °C, 48 h, 95% relative humidity, then oxygen-plasma treated	Ordered nanoporous thin film	Isopropanol	[102]
20		GQDs/SnO_2_/ ZnO	(1) ZnO nanorods: sputtering deposition of metal zinc and hydrothermal treatment of NH_3_·H_2_O + CMC + H_2_O at 120 ^o^C; (2) GQDs/SnO_2_/ZnO: Sn precursor solution (SnCl_4_ + GQDs + HCl) spin-coated onto ZnO nanorods, then a post-synthetic humidity treatment (95% relative humidity, 150 °C, then heated at 673 K for 2 h under N_2_)	Porous and hierarchical nanosheet heterostructure	H_2_S	[83]
21	Bimetallic oxide/GQDs composite	ZnFe_2_O_4_/ GQDs	(1) Hydrothermal treatment of GQDs + Zn(NO_3_)_2_·6H_2_O + Fe(NO_3_)_3_·9H_2_O + NaOH at 180 °C for 10 h	Nano-composites	Acetone	[111]
22		B-GQDs/ Ag-LaFeO_3_	(1) Ag-LaFeO_3_ (AL): sol–gel method + microwave treatment of La(NO_3_)_3_·6H_2_O + Fe(NO_3_)_3_·9H_2_O + citrate + AgNO_3_ + PEG for 2 h, then dried at 150 °C and heated at 800 °C for 2 h; (2) GQDs/Ag-LaFeO_3_: ultrasonicated, stirred and then dried of doped GQDs + AL	p-p heterojunction	HCHO	[109]
23		B-GQDs/ Ag-LaFeO_3_	(1) BI-ALs: benzene template + formaldehyde + AIBN + Ag-LaFeO_3_, ultrasonicated, polymerized, dried, and sintered; (2) B-GQDs/Ag-LaFeO_3_: microwave treatment of B-GQDs + BI-ALs for 2 h and dried at 80 °C overnight	p-p heterojunction	Benzene	[108]
24	Metal phthalocyanine (MPc)/GQDs	CoPc/GQDs	(1) MPc: C_9_H_4_O_5_ + urea + CoCl_2_·6H_2_O + (NH_4_)_6_Mo_7_O_24_·4H_2_O; (2) MPc/GQDs: ultrasonicated and dropped of MPc + GQDs onto interdigital electrodes and dried at 60 °C for 2 h	Hybrid materials	NO_2_	[58]
25		CoPc/GQDs	(1) CoPc-HFIP: CoPc-COOH + HFIP + DCC + DMF; (2) CoPc-6FBPA: CoPc-COCl + 6FBPA + pyridine + DMF; (3) CoPc/GQDs: CoPc-HFIP/CoPc-6FBPA + GQDs	Hybrid materials	DMMP	[101]
26	Polymer/GQDs	N-GQDs/ PEDOT-PSS	(1) Sonication and stirring of N-GQDs + PEDOT-PSS	Nano-composites	Methanol	[112]
27		S, N-GQDs/ PANI	(1) Chemical oxidative polymerization of S, N-GQDs + aniline + HCl + APS	Flexible hybrid material	NH_3_	[67]
28	Metal oxide/ polymer/ GQDs composite	ZnO/ S, N-GQDs/ PANI	(1) ZnO: ZIF-8 (template) + room temperature precipitation of (Zn(NO_3_)_2_·6H_2_O + 2-Methylimidazole); (2) ZnO/S,N-GQDs/PANI: in situ polymerization (aniline + HCl + S, N-GQDs + APS + ZnO + NaOH)	Nanopolyhedra	Acetone	[120]
29		PANI/ N-GQDs/In_2_O_3_	(1) In_2_O_3_: electrospinning (PVP + In(NO_3_)_3_ + ethyl alcohol + DMF) + high-temperature calcination (800 °C for 3 h); (2) PANI/N-GQDs/In_2_O_3_: in situ chemical oxidative polymerization (N-GQDs + In_2_O_3_ + HCl + aniline monomer + ammonium persulfate)	Hollow nanofiber	NH_3_	[115]
30	Metal sulfide/GQDs composite	MoS_2_/rGO/ GQDs	(1) MoS_2_: hydrothermal treatment of sodium molybdate dehydrate + thiourea at 200 °C for 12 h; (2) MoS_2_/rGO: MoS_2_ + GO at 200 °C 12 h; (3) MoS_2_/rGO/GQDs: hydrothermal treatment of MoS_2_/rGO + GQDs at 200 °C for 12 h	3D hybrids	NO_2_	[122]
31	GQDs/SiNW	GQDs/SiNW	(1) SiNW: metal-catalyzed electroless etching method (cleaned Si wafers + etchant); (2) GQDs/SiNW: GQDs dropped onto SiNW array surface	Nanowire array	NO_2_	[105]

**Table 3:** Gas sensing performance of GQDs-based chemiresistive gas sensors.

No.	Target Gas	Sensing Materials		Response (%)	Res (s)/Rec (s)	LOD (ppm)	Conc. (ppm)	T (°C)	Ref.
1	HCHO	N-GQDs/SnO_2_		256 ^a^	NA	NA	100	60	[118]
2		N-GQDs/SnO_2_		361 ^a^	330/30	0.01	10	60	[116]
3		B-GQDs/Ag-LaFeO_3_		18 ^b^	23/30	NA	1	55	[109]
4		N-GQDs/TiO_2_/graphene foam		9.1	18/20	NA	0.1	150	[99]
5	Acetone	GQDs/Pt-SnO_2_		NA	5–10/10–15	1	10	RT	[98]
6		SnO_2_/GQDs		120.6 ^a^	17/13	0.1	1000	275	[103]
				1.3 ^a^	30/35		0.1		
7		ZnFe_2_O_4_/GQDs		13.3 ^a^	9/4	5	1000	RT	[113]
				1.2 ^a^	4/2		5		
8		GQDs/ZnO		15.2 ^a^	9/16	0.0087	1	320	[107]
9		ZnO/S, N-GQDs/PANI		11.56 ^c^	15/27	0.1	2	RT	[120]
				25.6 ^c^	22/30		5		
10	NH_3_	GQDs		−14.9 ^d^ (pH 5)	26/21	NA	10	RT	[68]
				5.9 ^d^ (pH 7)	27/72				
11		OH-GQDs		76.63 ^c^	64/69	NA	500	RT	[121]
12		ZnO/GQDs		6047 ^a^	NA	NA	1000	RT	[106]
				760 ^a^	NA	NA	100		
13		S, N-GQDs/PANI		42.3 ^d^	115/44	0.5	100	25	[67]
				385 ^d^			1000		
14		PANI/N-GQDs/In_2_O_3_		15.6 ^b^	NA	NA	1	RT	[115]
15	H_2_S	GQDs/SnO_2_/ZnO		15.9^a^	14/13	NA	0.1	RT	[83]
16		Co, N-GQDs/SnO_2_		37.3 ^a^	5/11	0.05	100	260	[119]
17	Isopropanol	GQDs/TiO_2_		13.8	18/11	NA	50	RT	[123]
18		PtO_x_/GQDs/TiO_2_		4.4 ^c^	9/ NA	NA	1	RT	[102]
19	Methanol	N-GQDs/PEDOT-PSS		13.4 ^d^	12/32	NA	100	RT	[112]
20	Trimethylamine	GQDs/α-Fe_2_O_3_		1033 ^a^	11/24	0.01	1000	270	[84]
				1.9 ^a^	6/4		0.01		
21	Acetic acid	GQDs/ZnO		642 ^a^	43/49	1	1000	RT	[110]
				1.1 ^a^	11/12		1		
22	Benzene	B-GQDs/Ag-LaFeO_3_		17.5 ^b^	NA	1	1	65	[108]
23	DMMP	CoPc/GQDs	CoPc-HFIP/ GQDs	8.4 ^d^	600/640	0.5	20	RT	[101]
			CoPc-6FBPA/ GQDs	9.3 ^d^	600/620				
24	NO_2_	GQDs/SiNW		140 ^e^	NA	10	500	RT	[105]
25		N-GQDs/SnO_2_		292 ^b^	181/81	0.02	0.1	150	[118]
				4336 ^b^	528/384			50	
26		N-GQDs/In_2_O_3_		81.7 ^b^	95/36	0.1	1	100	[70]
27		N-GQDs/ZnO		58 ^b^	NA	0.1	5	100	[114]
28		N-GQDs/SnO_2_		417 ^b^	59/33	0.1	1	130	[100]
39		CoPc/GQDs		15.8 ^a^	100/100	0.05	50	RT	[58]
30		Fe_3_O_4_-rGOQD- naphthalene-2-SO_3_H		130 ^d^	25/97	NA	50	RT	[104]
31		MoS_2_/rGO/GQDs		23.2 ^d^	150/150	5	50	RT	[122]
				15.2 ^d^			5		
32	NO	TiO_2_/N-GQDs		12 ^d^	298/890	NA	100	RT	[113]
				31.1 ^d^	235/285			RT+UV	

Res(s)/Rec(s) stands for Response time (s)/Recovery (s); LOD stands for Limit of detection; Conc. stands for concentration; T stands for Temperature; NA stands for not applicable; ^a^ indicates that *R_a_*/*R_g_*; ^b^ indicates that *R_g_*/*R_a_*; ^c^ indicates that (*R_a_* − *R_g_*)/*R_a_* × 100%; ^d^ indicates that (*R_g_*− *R_a_*)/*R_a_* × 100%; ^e^ indicates that (*I_g_ − I_a_*)/*I_a_* × 100%.

## Data Availability

Not applicable.
